# Molecular modeling of highly selective CDK1 Inhibitors based on pyrazolo-pyrimidines using 3D-QSAR, docking, and molecular dynamics simulations

**DOI:** 10.1371/journal.pone.0350566

**Published:** 2026-06-22

**Authors:** Muhammad Afaq Tahir, Tahir Ali Chohan, Aisha Qayyum, Abdullah Yahya Abdullah Alzahrani, Abdullah R. Alzahrani, Zia Ur Rehman, Abida Khan, Khuram Ashfaq

**Affiliations:** 1 Institute of Pharmaceutical Sciences, university of veterinary and Animal Sciences, Lahore, Pakistan; 2 Department of Pharmacy, Faculty of Pharmaceutical Sciences, Green International University, Lahore, Pakistan; 3 Department peadiatric medicine, Fatima memorial hospital, Lahore, Pakistan; 4 Department of Chemistry, Faculty of Science, King Khalid University, Abha, Kingdom of Saudi Arabia; 5 Department of Pharmacology and Toxicology, Faculty of Medicine, Umm Al-Qura University, Al-Abidiyah, Makkah, Saudi Arabia; 6 Health Research Centre, Jazan University, Jazan, Saudi Arabia; 7 Department of Pharmaceutical Chemistry and Pharmacognosy, Faculty of Pharmacy, Jazan University, Jazan, Kingdom of Saudi Arabia; 8 Center For Health Research, Northern Border University, Arar, Saudi Arabia; 9 Faculty of Pharmaceutical sciences, Lahore university of biological and Applied Sciences, Lahore, Pakistan; University of Nairobi Faculty of Health Sciences, KENYA

## Abstract

Cyclin-dependent kinase 1 (CDK1) has emerged as a compelling target for anticancer drug development due to its essential role in cell cycle regulation. In this study, a series of pyrazolopyrimidine-based inhibitors was investigated through an integrated computational approach that combined molecular docking, three-dimensional quantitative structure-activity relationship (3D-QSAR) modeling, and molecular dynamics simulations. CoMFA and CoMSIA models were established to elucidate the structural features influencing CDK1 inhibition, demonstrating high reliability with q² and r² values of 0.58 and 0.945, respectively. Docking studies using the crystal structure of CDK1 (PDB ID: 4Y72) revealed key interactions and hotspot residues, such as L83, V18, and D86, which contribute significantly to ligand binding. From a library of 100 in-house compounds, compounds 34 and 37 exhibited strong binding affinities (−12.61 and −12.50 kcal/mol) and were further evaluated through molecular dynamics simulations. The stability of these complexes was supported by RMSD analysis and binding free energy calculations (−94.99 kcal/mol for compound 34). Moreover, ADMET profiling indicated favorable pharmacokinetic properties and synthetic accessibility. These findings offer critical insights into the structural determinants of CDK1 inhibition and support the further development of pyrazolopyrimidine scaffolds as potential anticancer agents.

## 1. Introduction

Cancer persists as a major global health issue, influencing the lives of millions around the world. Cancer treatment has always been regarded as one of the most critical and vital aspects of clinical challenges [1–3]. Advancements in understanding cancer pathways, coupled with enhanced technological platforms, have led to the development of precision therapeutics that specifically target aberrant cancer pathways, significantly improving patient outcomes [4]. Cell cycle dysregulation is a critical factor in the onset and progression of cancer, and it is a hallmark characteristic of tumor cells [5]. The cell cycle comprises specific phases: the G1 and G2 phases, during which cells prepare for proliferation; the S phase, where DNA replication occurs; and the M phase, which involves mitosis and cell division [6,7]. The cell cycle progresses through the sequential activation and inactivation of specific cyclin-dependent kinases (CDKs) during stages such as G0, G1, S, G2, and M phases [8]. These CDKs are divided into two main groups: cell-cycle CDKs (CDK1, CDK2, CDK4, and CDK6), which are essential for cell cycle advancement, and transcriptional CDKs (CDK7, CDK8, and CDK9), which play a key role in the transcription process [9,10]. Among the CDK family, CDK1 stands out as a master regulator of the cell cycle [11]. Hence targeting CDK1 in the cycle can prove critical to stop cell cycle progression [12,13]. The mechanistic role of CDK1 in cell cycle regulation, including schematic aspects of its inhibition, has been previously reported by us in an earlier review article and is provided again in the Supporting Information (**S1 Fig in** S1 File) to aid reader understanding [14].

The development of compounds specifically targeting CDK1 and inhibiting its activity can effectively stop cells from progressing into mitosis, acting as a crucial factor for inducing apoptosis, which leads to programmed cell death [15,16]. This mechanism of CDK1 inhibition has shown great success in cancer cells where CDK1 is overexpressed, driving continuous and uncontrolled cell division a hallmark of cancer [17,18]. Additionally, CDK1 inhibition not only halts cell cycle progression but also reduces the activity of certain proteins that promote cancer stem cell (CSC) characteristics, which play a significant role in tumor growth and resistance [19]. By targeting CSCs and limiting their activity, CDK1 inhibitors disrupt tumorigenic behavior, improving cancer treatment outcomes, particularly in aggressive cancers such as pancreatic ductal adenocarcinoma [20]. CDK1 typically forms a complex with Cyclin B, essential for the transition from the G2 phase to the M phase of the cell cycle [21]. By inhibiting CDK1, these compounds block the phosphorylation of proteins necessary for mitotic progression, arresting the cell cycle in the G2 phase. This leads to cell cycle arrest and often triggers apoptosis in cancer cells. In cancers characterized by rapid cell division, CDK1 inhibitors leverage this vulnerability, effectively slowing or stopping cancer proliferation and inducing cell death, making them a promising therapeutic approach in oncology [22].

Molecular modeling simulations have been utilized as a powerful approach in the design of CDK1 inhibitors [17,23]. In particular, three-dimensional quantitative structure-activity relationship 3D-QSAR models have been created to statistically evaluate the structure-activity relationships (SAR) of known CDK1 inhibitors, employing techniques such as comparative molecular field analysis (CoMFA) and comparative molecular similarity indices analysis (CoMSIA) [24–27]. Recently, **pyrazolopyrimidine** derivatives have been synthesized and evaluated to be active inhibitors of CDK1. To gain a deeper understanding of the pharmacological properties of these compounds and to search for more potential CDK1 inhibitors, this study conducted a comprehensive computational analysis of 50 pyrazolopyrimidine derivatives [28–34]. To study the relationship between molecular structure and activity some different computational techniques, such as 3D-QSAR, molecular docking simulations, molecular electrostatic potential (MESP) analyses, molecular dynamics (MD) simulations, and MMPBSA19/MMGBSA20 binding free energy calculations were comprehensively studied. 3D-QSAR employed molecular alignment-based methodologies to statistically establish a correlation between the biological activity of a series of compounds and the spatial characteristics surrounding these molecules, including steric, electrostatic, and hydrophobic properties, as well as hydrogen bond donor and acceptor regions [35,36]. To establish a theoretical framework for the discovery of novel CDK1 inhibitors with a shared parent heterocyclic core, we designed a series of innovative CDK1 inhibitors exhibiting strong predicted potency. Herein, a combination of Molecular Electrostatic Potential (MESP) analysis and molecular docking techniques has been effectively utilized to elucidate the influence of various interactive fields and structural prerequisites essential for highly potent CDK1 inhibitors. The binding stability of these inhibitors to CDK1 was further confirmed through molecular dynamics simulations, accompanied by the calculation of their binding free energy. The findings of this study contribute to identifying crucial structural and pharmacophoric features influencing the binding mechanism, offering valuable insights for the rational design of novel, potent, and selective CDK1 inhibitors through targeted structural modifications.

## 2. Computational methods

### 2.1. Dataset for 3D-QSAR analyses

A total of 50 compounds featuring the pyrazolopyrimidine core were identified and gathered through a comprehensive literature review, with corresponding structural and activity data obtained for analysis [28–34]. The dataset size is consistent with commonly reported CoMFA and CoMSIA studies, where datasets typically range between 40 and 70 compounds [26,37]. The reported IC₅₀ values were converted to pIC₅₀ values (pIC₅₀ = −log₁₀ IC₅₀) to ensure uniform activity scaling for QSAR modeling. For consistency, all QSAR modeling and statistical analyses were performed using pIC₅₀ values. IC₅₀ values are reported in selected sections solely to facilitate intuitive biological interpretation, without affecting the consistency of the modeling framework. The dataset was partitioned into a training set (38 compounds) and a test set (12 compounds), corresponding to an approximate 75:25 ratio. This partitioning strategy is widely employed in QSAR modeling, as it provides sufficient data for model development while maintaining an independent dataset for external validation [37]. The division was performed using a random partitioning approach, followed by evaluation to ensure that both subsets adequately represented the range of biological activity and structural diversity within the dataset. The predictive reliability of the developed QSAR models was assessed using standard statistical validation parameters and further supported by evaluation against the independent test set, demonstrating consistent predictive performance across the activity range.

#### 2.1.1. Preparation of compounds and structural optimization.

The 3D structures of the 50 compounds were generated in the SYBYL-X2.0 molecular simulation suite using standard geometric parameters within its drawing module [38]. To minimize the compounds’ energy, Gasteiger-Hückel charges, and the Tripos force field were applied, followed by optimization using the Powell energy gradient method. [39] This process was conducted with a maximum of 100,000 iterations and an energy convergence criterion of 0.05 kcal/mol, while other settings were maintained at default values. Structural alignments were performed by superimposing the pyrimidine core of each compound onto the experimentally determined bioactive conformation of compound 27g, targeting CDK1. Compound 27g was selected as the alignment template due to its high inhibitory activity within the dataset and its ability to represent the key structural and pharmacophoric features of the pyrazolopyrimidine scaffold. The use of a highly active and structurally representative compound as a reference ensures that the alignment captures the essential steric and electrostatic characteristics required for effective CDK1 inhibition, thereby improving the reliability and interpretability of the resulting CoMFA and CoMSIA models. These alignments served as the foundation for CoMFA and CoMSIA analyses in subsequent model development, as precise molecular alignment is critical to our objectives; the alignment results significantly influence the model’s accuracy and predictive reliability [40–42].

#### 2.1.2. QSAR model creation.

3D-QSAR is a research methodology that integrates traditional QSAR with the three-dimensional structural characteristics of compounds, providing a robust framework for investigating drug-receptor interactions, deducing receptor architectures, establishing structure-activity relationships, and optimizing drug candidates. This approach primarily encompasses Comparative Molecular Field Analysis (CoMFA) and Comparative Molecular Similarity Index Analysis (CoMSIA) [43,44]. Both CoMFA and CoMSIA are widely employed to design templates for synthesizing novel drugs and to elucidate the key chemical interactions governing target behaviors. In these methods, aligned molecules are positioned within a three-dimensional grid with regularly spaced points. In this study, a grid spacing of 2.0 Å was used for both CoMFA and CoMSIA analyses. In CoMFA, steric (S) and electrostatic (E) fields serve as descriptors, calculated using a probe such as an sp³-hybridized carbon atom with a + 1.0 charge. CoMSIA expands upon this by incorporating additional fields, including hydrophobic (HP), hydrogen bond donor (HBD), and acceptor (HBA) properties. The electrostatic field in CoMFA is determined by the Coulomb function, while the steric field relies on the Lennard-Jones function. In contrast, CoMSIA utilizes a Gaussian function with distance-dependence to calculate molecular fields, enabling the use of more nuanced similarity metrics between grid points. The molecules are enclosed within a rectangular box, and the molecular fields within this lattice act as independent variables in QSAR modeling. To enhance computational efficiency and reduce noise, a column filter threshold of 2.0 kcal/mol is applied to reduce noise and eliminate insignificant variables, thereby improving model robustness and statistical reliability, as commonly practiced in CoMFA and CoMSIA studies [45]. The model’s predictive capacity is evaluated using the correlation coefficient, R²pred, calculated by summing the squared variances of the predicted and actual activities for the test set compounds.


$$\begin{array}{c} r_{{pre}^{\text{2}}}=\left(\frac{SD-PRESS}{SD}\right) \end{array}$$

This allows for a robust assessment of the model’s capability to predict the biological activity of novel compounds [46]. The internal validation and statistical quality of the developed QSAR models were further evaluated using standard parameters, including the cross-validated correlation coefficient (q²), non-cross-validated correlation coefficient (R²ₙcv), standard error of estimate (SEE), and F-statistic. The cross-validated coefficient (q²) is a key parameter that reflects the internal predictive ability and robustness of the model, indicating how well the model can predict unseen data within the dataset. These parameters were used to assess the robustness, predictive ability, and statistical significance of the models.

### 2.2. Novel CDK1 inhibitors using *in-silico* approach

The 3D-QSAR studies conducted on these CDK1 inhibitors, particularly the prime compound 27g, provided valuable structural insights for the computational design of a library of 100 in-house compounds using Spark™ by Cresset https://www.cresset-group.com/ software/spark/ [47]. Spark excels in simultaneously exploring electrostatic and shape space, offering superior molecular compatibility compared to other tools. This software allowed for enhanced flexibility when expanding ligands into unoccupied spaces or linking fragments from different regions of a protein active site [48]. For compound design, we utilized an in-house library of chemical fragments, selecting the optimal compound based on IC₅₀ values and contour maps. Consequently, a library of 100 novel CDK1 inhibitors was computationally generated, leveraging Spark’s R-group exploration strategy with the top compound, 27g, serving as the prototype. The design of these compounds was guided by systematic R-group modification of compound 27g, informed by the structural features identified in the CoMFA and CoMSIA contour maps. Favorable regions corresponding to steric, electrostatic, hydrophobic, and hydrogen bonding interactions were specifically targeted to introduce substituents that could enhance binding affinity and biological activity. During lead optimization, Spark effectively suggested new compounds derived from the accessible chemical space, aiding in the selection of candidates for synthesis. The compounds were then mapped onto the 3D-QSAR model for activity prediction. A complete structural and activity data of these 100 compounds named from 1–100 has been provided in Table S2 in S1 File in the supporting information.

### 2.3. Molecular docking

The molecular docking approach is a crucial tool for exploring the binding interactions between small molecules and proteins, which aids in elucidating the pharmacological mechanisms of potential drug candidates and facilitates the discovery of new therapeutic options [49]. For this study, docking simulations were conducted using the crystal structure of the CDK1 complex with a co-crystalized pyrazole-based inhibitor (PDB ID: 4Y72). The protein preparation involved removing all co-crystallized water molecules using the Protein Preparation Wizard in Schrodinger Suites 2020_3 × 64 [49]. Missing side chains and loops were incorporated via Prime, while PROPKA was applied to adjust the protonation and tautomeric states of ASP, GLU, ARG, LYS, and HIS at pH 7.4 [50]. The water molecules beyond 5.0 Å from the ligand were excluded, followed by conformational optimization and minimization. Ligand molecules were prepared for docking using LigPrep, which generated various structures by adjusting ionization states, tautomers, stereochemistry, and ring conformations [51,52]. Each ligand was optimized with the OPLS4 force field to achieve the lowest energy conformation [53]. The glide grid was set within a 10 Å radius of the co-crystallized ligand, targeting ligand-binding sites. This search space, defined by the Glide receptor grid, encompassed the protein’s force field information. The 150 including the 100 inhouse ligands were docked into the CDK1 binding site using Glide in Schrodinger Suites 2020_3 × 64, with the protein structure and receptor grid selected in the Glide panel and the prepared ligand structures as input. The docking was conducted in Glide XP mode, and results were analyzed with Schrodinger’s Maestro interface and visualized using PyMOL for detailed protein-ligand interaction insights [54–56].

### 2.4. Binary interaction fingerprinting

The binary interaction fingerprinting is important in determining the residual interaction between proteins and the docked ligands. It provides a detailed three-dimensional interaction pattern between all the interacting residues within the receptor site of the protein [57,58]. This interaction fingerprinting data enables us to analyze the binding characteristics of a vast number of ligands within the same binding pocket of a protein. Binary codes symbolizing “0” and “1” are used to represent the absence or presence of interaction with a particular residue residing within the binding pocket respectively. The enormous amount of information found in ligand-receptor complexes can be arranged, examined, and presented using fingerprints.

### 2.5. MD Simulation studies

Molecular dynamics simulations have been performed on ligand-protein complexes for selected complexes CDK1-27g, CDK1-27l, and the model obtained compounds CDK1–37 and CDK1–34 from the designed library. The main purpose of performing MD simulation was to determine the chemical stability of the compounds bound to the protein using complexes [59,60]. Initially, the LEaP system was applied to the protein and ff14SB amber parameters were applied to the protein and the ligands simultaneously [61,62]. Solvation of the complex was ensured using SPCBOX at a marginal distance of 9A and the PDB format of this solvated complex was saved. LEaP system again produced a parameter as well as a coordinated file of the PDB and the minimization of the compound was done thrice to remove steric hindrance. After completing the minimization step the heating module was applied to system and a gradual increase in temperature took place starting from 0K to ending at 300K. At 300 K equilibration procedures were performed to further stabilize the system.MD simulation was performed following the previous methods reported by us at 100 ns utilizing the NPT ensemble provided the conditions 300K and 1atm pressure [57]. Finally, the generated MD simulation results were analyzed, including parameters such as root mean square deviation (RMSD), root mean square fluctuation (RMSF), radius of gyration (Rg), and further analysis of the protein-ligand complex was performed. Hydrogen bond analysis was conducted throughout the MD trajectories using standard geometric criteria (donor–acceptor distance ≤ 3.5 Å and angle ≥ 120°) to evaluate the stability of protein-ligand interactions.

### 2.6. Binding free energy calculations by MMPBSA/MMGBSA and energy decomposition analysis

To evaluate the binding affinity between the protein and the molecule, and to determine the stable molecular conformation and binding free energy, we analyzed the equilibrium trajectory from the last 20 ns of the simulation [63]. The MM/PBSA algorithm was employed to assess the trajectory records from the MD simulation and calculate the binding energy for various ligands bound to the crystal complex [64,65]. Furthermore, the free energy landscape was utilized to monitor the binding interactions between the proteins and compounds, providing insights into the stability tendency of the protein complex [66].

### 2.7. ADME analysis and toxicity prediction

The ADMET properties of the newly designed compounds were evaluated using SwissADME (http://www.swissadme.ch), which helped assess the synthetic accessibility of these compounds. For further computer-aided ADME studies, the pkCSM webserver (http://biosig.unimelb.edu.au/pkcsm/prediction) was used to predict the pharmacokinetic characteristics of our designed compounds. This analysis covered various parameters, including intestinal absorption, volume of distribution (VDss), and metabolism through cytochrome (CYP) binding, excretion with total clearance values, AMES toxicity prediction, and synthetic accessibility using SwissADME (http://www.swissadme.ch/index.php) [67].

## 3. Results & discussion

### 3.1. 3D QSAR analysis

The **Pyrazolo-pyrimidine derivatives** described in recent studies were used to develop the QSAR model. Table 1 illustrates the statistical parameters derived using CoMFA and CoMSIA, respectively. To facilitate clearer interpretation of the structure-activity relationships, a summary of representative compounds along with their corresponding pIC₅₀ values and key QSAR insights is presented in Table 2. The results presented by the CoMFA model (Table 1) demonstrate q^2^ = 0.58, ONC = 5, R²ₙcv = 0.945, SEE = 0.246 and F = 116.704. The results of the model’s steric and electrostatic fields indicated that the electrostatic contribution was much bigger (53.8%) than the steric effect (46.2%). The CoMSIA model describes the effectiveness of the QSAR model via an analysis of the five parameters; steric (S), electrostatic (E), hydrophobic (H), hydrogen bond donor (D), and hydrogen bond acceptor (A). The CoMSIA values compare and distinguish the contributions made by each field. The CoMSIA model produced the q^2^ value of 0.53, ONC = 6, the R²ₙcv value of 0.967, the SEE value of 0.195, and the F value of 135.54. The CoMSIA model’s field contribution showed H-bond donor and acceptor field values of 24.4% and 11.4%, respectively, in addition to 9%, 27.8%, and 27% for the steric fields, hydrophobic, and electrostatic steric fields. The findings demonstrate that chemical compounds play a crucial role in hydrogen bonding for ligand-protein complexes, whereas electrostatic and hydrophobic field contributions may contribute to better ligand binding. The test set validation of QSAR model data from q^2^ and R²ₙcv supports establishing a credible prediction model to evaluate the activity of covalent compounds inhibiting CDK1. The external predictive performance of the developed QSAR models was further supported by the predictive correlation coefficient (R²pred), indicating good agreement between predicted and experimental activities of the test set compounds. The applicability domain of the developed QSAR model was further evaluated using a Williams plot and Tanimoto similarity-based clustering **S2 Fig in** S1 File. The results confirm that the majority of compounds fall within the acceptable limits and are well distributed within the chemical space defined by the training set, supporting the reliability of the model predictions.

**Table 1:** Statistical parameters from CoMFA and CoMSIA models for CDK-1.

	CDK1	
**PLS Statistics**	**CoMFA**	**CoMSIA**
*q* ^*2a*^	0.58	0.53
*ONC* ^*b*^	5	6
*R*_*ncv*_^*2c*^	0.945	0.967
*Probability of r* ^*2*^	0.000	0.000
*SEE* ^*d*^	0.246	0.195
*F* ^*e*^	116.704	135.54
*R*_*pred*_^*2f*^		0.969
**Contributions**		
*Steric*	0.462	0.094
*Electrostatic*	0.538	0.270
*Hydrophobic*		0.278
*H-bond donor*		0.244
*H-bond acceptor*		0.114

*q²ᵃ: cross-validated correlation coefficient (leave-one-out method);*

*ONCᵇ: optimal number of components;*

*R²ₙcvᶜ: non-cross-validated correlation coefficient;*

*SEEᵈ: standard error of estimate;*

*Fᵉ: F-test value indicating the statistical significance of the model;*

*R²preᶠ: predictive correlation coefficient for the test set.*

**Table 2:** Representative compounds with pIC₅₀ values and key QSAR insight.

Compound	pIC₅₀	Key QSAR Insight
**27g**	High	Optimal electrostatic and H-bond interactions; reference compound
**27f**	High	Favorable electrostatic contribution at R2
**27e**	Moderate	Increased steric bulk reduces activity
**27i**	Moderate	Lack of bulky groups improves steric compatibility
**27k**	High	Favorable hydrogen bond acceptor contribution
**27l**	Low	Unfavorable electrostatic and steric interactions

**Fig 1:**
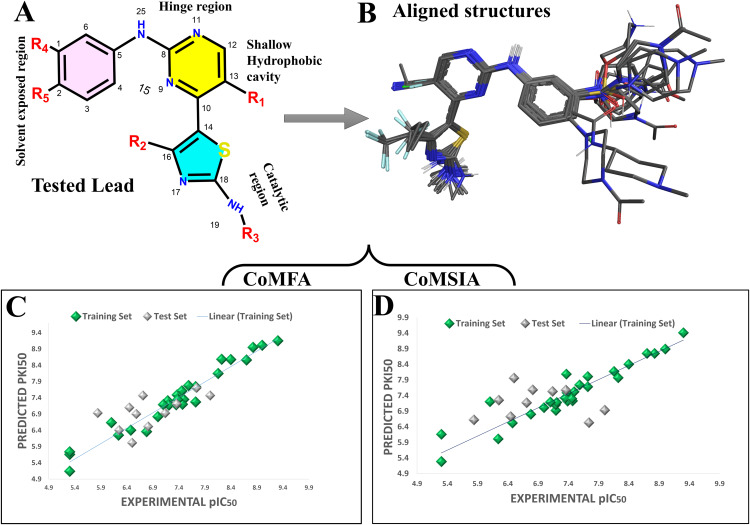
(A) The basic compound with core ring where QSAR alignment was made. (B) Final alignment for training and test set molecules. (C) CoMFA and (D) CoMSIA model predicted pIC₅₀ values against experimental values.

The correlation between the experimentally observed pIC₅₀ and the predicted pIC₅₀ values, generated through CoMFA and CoMSIA models using both the training and test datasets, is depicted as a scatter plot in Fig 1C and 1D. The plot reveals a linear trend, with the majority of compounds positioned close to the line, indicating a strong relationship between the observed and predicted pIC₅₀ values. This alignment supports the validity of the models in accurately predicting the CDK1 inhibitory potential of the compounds.

#### 3.1.1. CoMFA analysis of CDK1 inhibitors.

**Fig 2:**
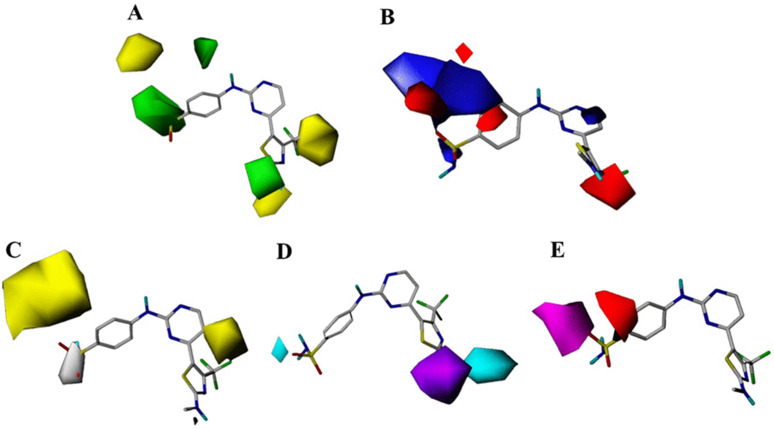
CoMFA contour maps (A and B) and CoMSIA (C, D, and E) contour maps for CDK1 were presented under compound 27g. Steric **(A)**; green contours demonstrate the bulky groups needed to rationally select molecules with high activity, while yellow contours disfavor this acquisition. Electrostatic **(B)**; blue contours illustrate the requirement of positively charged groups for the region, while red contours indicate the presence of negatively charged groups needed for sufficient activity. Hydrophobic **(C)**; yellow contours signal beneficial areas for activity, while white contours oppose this direction. H-bond donor **(D)**; cyan contours indicate the need for hydrogen bond donor groups, while purple contours illustrate the disfavored regions for such groups. H-bond acceptor **(E)**; magenta contours represent favored sites for hydrogen bond acceptor groups, while brown contours indicate regions where these groups negatively impact activity.

Fig 2 represents contour maps of COMFA fields generated with the highly bioactive compound **27g** regarding CDK1. For clarity in discussing biological potency, IC₅₀ values are occasionally referenced in the contour map interpretation, while pIC₅₀ values were consistently used for QSAR model development and statistical analysis. In the CDK1 CoMFA steric contour map (Fig 2A), the green contours indicate steric bulk favorable sites for CDK1b inhibitors, while the yellow contours suggest regions where bulky groups are unfavorable. Specifically, the presence of bulky groups is not favorable at the R2 and R3 positions on ring A. This trend is evident in compounds **27e** and **27f,** which share all chemical moieties except for R2. Compound **27f**, with a trifluoride group at R2, shows an inhibitory value of 1.5 nM. However, the addition of a methyl group to this trifluoride group in **27e** significantly decreases its activity by over a hundredfold. Moreover, compound **27b**, with a trifluoromethyl group at R2 of ring A, exhibits almost no inhibitory value. On the other hand, the lack of bulky groups in compound **27i** correlates with a potent inhibitory value of only 4 nM. These steric considerations provide useful insights for designing new compounds that avoid bulky groups in specific areas. Additionally, the large green contour around ring C is supported by compounds like **12k** and **12l,** which possess 4-acetylpiperazin-1-yl and piperazin-1-yl groups at the R5 position, respectively. These compounds demonstrate significant activity compared to compounds lacking steric bulk, such as **9i**, which only has a methyl group and shows nearly tenfold lower activity.

Fig 2B represents the electrostatic contours of the CoMFA model for CDK1 inhibitors. The red contours surrounding ring A indicate areas where negative groups are favorable, which is exemplified by the best compound from our model, **27g**, containing a trifluoromethyl group at the R2 position of ring A. This compound exhibits high bioactivity with an IC₅₀ value of 0.5 nM. On the other hand, the presence of red contours also suggests that the inclusion of any positive groups at this site is unfavorable. This observation was validated by another compound, **27i**, where the presence of just a proton at the same position resulted in a more than eightfold reduction in inhibitory activity. Similarly, the red contours around ring C support the presence of negatively charged groups. This trend is supported by compound **27k**, which has a sulfur dioxide group at the R5 position of ring C, leading to a significant inhibitory potential with an IC₅₀ value of 2 nM. In contrast, compound **27l**, which shares a similar structure to **27k** but with a positively charged 1,4-diazepan-1-yl group at the R4 position, exhibited a sharp decline in activity, reducing its potency by nearly ninetyfold.

Moreover, the blue contours surrounding ring C at the R4 position favor the presence of positively charged groups. This is observed in compound **27f,** where a primary amine group at the -R4 position demonstrated a strong inhibitory activity with an IC₅₀ value of 1.5 nM. In contrast, the introduction of a negatively charged group at the same position, as seen with the hydroxyl group in compound 27c, resulted in a drastic reduction in activity by around 370 times. Additionally, another blue contour is observed on the opposite side of this region, which further supports the presence of electropositive atoms. This is evident from the rotatable attachment of the primary amine group at positions R4 and R5, indicating that its rotation leads to the formation of this contour, favoring the presence of positively charged groups in that region.

#### 3.1.2. CoMSIA analysis of CDK1 inhibitors.

Fig 2C-2E depict the contour maps of CoMSIA fields (Hydrophobic, H-bond donor, and H-bond acceptor) maps around compound **27g**, which exhibits the highest activity for CDK1 inhibition. As shown in Fig 2C both the presence and lack of hydrophobic groups at specific locations are important for CDK1 inhibitory activity. The yellow contours indicate regions where hydrophobic groups are favorable in the most optimal compounds. The contour located along the R1 and R2 positions suggests that the presence of hydrophobic groups is advantageous. This is supported by compound **1a**, which has a methyl group at the R2 position. The presence of this hydrophobic group allows 1a to achieve an inhibitory value of 5 nM. However, replacing the methyl group with a trifluoromethyl group in compound **27e** significantly reduced its activity, leading to a thirtyfold decrease in inhibition. Furthermore, the presence of a large hydrophobic contour parallel to ring C is validated by compound **9j**, which demonstrated fifty times better activity compared to compound **9p**, which lacks any substituent at the R5 position of ring **C.** The white contour between the R4 and R5 positions indicates that hydrophilic groups are favorable in this region, as supported by our best-performing compound **27g**, which has a sulfonamide group at this site. This hydrophilic group contributes to the high inhibitory activity of the compound. On the other hand, the presence of hydrophobic groups in this region is unfavorable, as demonstrated by compound **9f**, which exhibited sixty times lower activity compared to **27g** due to its hydrophobic substituent.

In Fig 2D, the CoMSIA-generated hydrogen bond donor map, represented by cyan and purple contours, illustrates how hydrogen bond donor substituents affect the binding affinity of CDK1 inhibitors. The cyan contours highlight regions where the addition of a hydrogen bond donor group is favorable, while the purple contours indicate that adding a hydrogen bond donor in those areas would reduce the inhibitory activity. For instance, compound **9a** has a primary amine attached to ring A due to the presence of hydrogen at the R3 position. This configuration enhances the inhibitory potential, achieving an IC₅₀ value of 7 nM. However, when this group is modified from a primary amine to a secondary amine by adding a methyl group at R3, a significant reduction in activity is observed. This trend is evident in compounds like **9k** and **9p**, where the change in the substituent leads to a reduction in activity ranging from over ninetyfold in **9k** to nine hundredfold in **9p**. Furthermore, if the alkyl group on the secondary amine is replaced by a methyl group, the addition of a hydrogen-donating group becomes unfavorable, as indicated by the purple contours. This unfavorable interaction is reflected in compounds like **12b** and **12d**, both of which exhibit a marked decrease in activity due to the introduction of this unfavorable group.

In Fig 2E, the hydrogen bond acceptor region is illustrated by magenta and green contours. The magenta areas signify favorable positions for hydrogen bond acceptor groups, while the red contours indicate that hydrogen bond acceptors at those positions would reduce activity. The formation of this contour is confirmed by compound **27h**, which contains a sulfur dioxide group at the R5 position of ring C. This compound exhibits strong activity with an IC₅₀ value of 1 nM, supporting the presence of a hydrogen bond acceptor at this site. However, the absence of a hydrogen bond acceptor from this position leads to a significant reduction in activity. This is exemplified by compound **27j**, where the hydrogen bond acceptor group is moved from the R5 to the R4 position, resulting in an 18 fold decrease in inhibitory activity.

On the other hand, the red contour indicates disfavored regions for hydrogen bond acceptors, especially parallel to the R5 position of ring C. This unfavorable interaction is validated by compound **12l**, which contains a piperazine-1-yl group around ring C and demonstrates reduced activity, showing an IC₅₀ value of only 45 nM. The compounds cited here serve as precise examples that validate the contours generated by our model.

### 3.2. Computational design of novel CDK1 inhibitors

Computational methods using SPARK software were employed to generate a virtual chemical library and produce new CDK1 inhibitors. Applying the R-group technique, **100** new compounds were created and further evaluated through docking studies using Schrodinger Suites software. The details of these compounds can be found in **Table S2 in** S1 File in the supplementary information. All the generated compounds showed comparable results, with each compound achieving a glide score greater than −6 kcal/mol. Among them, compounds **34 and 37** exhibited the best results and were subsequently subjected to MD simulation to assess their binding mode, interaction profile, and complex stability.

### 3.3. Interaction pattern analysis using structural interaction fingerprinting and molecular docking approach

**Fig 3:**
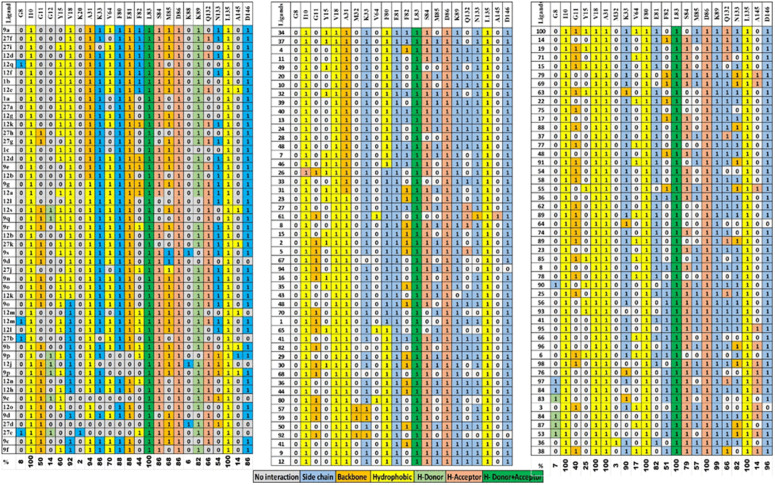
Interaction fingerprint: Protein-ligand interaction fingerprinting for hits retrieved from the Schrodinger Suite 2023 library at residues engaged in the interaction site within 4.0 Å of the ligands. The numerals 1 and 0 represent the presence and absence of interactions with residues, respectively. For residue coloring, hydrophobic, H-bond donor, H-bond acceptor, and backbone properties are utilized. Residues with an occupancy rate greater than 90% are considered essential for ligand binding. The percentage occupancy rate for each residue is calculated and displayed at the bottom of the table.

In this study, we utilized structure-based techniques to delve into the structural relationships elucidated by the ligand-based 3D-QSAR model, enhancing our comprehension of the molecular interactions between potential CDK1 inhibitors and the CDK1 protein. The computational model effectively identified crucial molecular patterns essential for creating inhibitors with strong binding affinities to CDK1. A total of 150 ligands were initially screened using the Glide module from Schrödinger Suites 2020, allowing for a precise evaluation of interaction patterns. For each docked ligand, a binary structural interaction fingerprint was generated, capturing intricate details of side-chain interactions, backbone contributions, hydrophobic forces, and hydrogen bond (H-bond) interactions, including donor, acceptor, and donor-acceptor features. These collective interaction patterns offered insights into the binding requirements for potent CDK1 inhibitors. A detailed representation of the interactions between CDK1 and the ligands is depicted in Fig 3 illustrating that among the 50 top-ranked ligands, nearly all formed interactions with key residues, notably I10, V18, A31, L83, and L135. Conversely, in a set of 100 in-house compounds, additional residues were involved in binding, with the majority interacting with residues I10, V18, A31, F80, L83, D86, K89, L135, and D146. This pattern highlights I10, V18, A31, L83, and L135 as “hot-spot” residues, indispensable for effective binding across both compound series. Among these, L83 emerged as particularly significant, functioning as both an H-bond donor and acceptor for all 150 ligands, suggesting its potential for further exploration in structure-activity relationship studies aimed at optimizing CDK1 inhibitors. Additional H-bond interactions involved residues I10, S84, D86, Q132, and A145 as acceptors, while G8, G12, and K89 acted as donors. Notably, residues I10, Y15, V18, A31, V64, F80, F82, L135, and A145 predominantly contributed to hydrophobic interactions, underscoring their role in stabilizing ligand binding.

**Fig 4:**
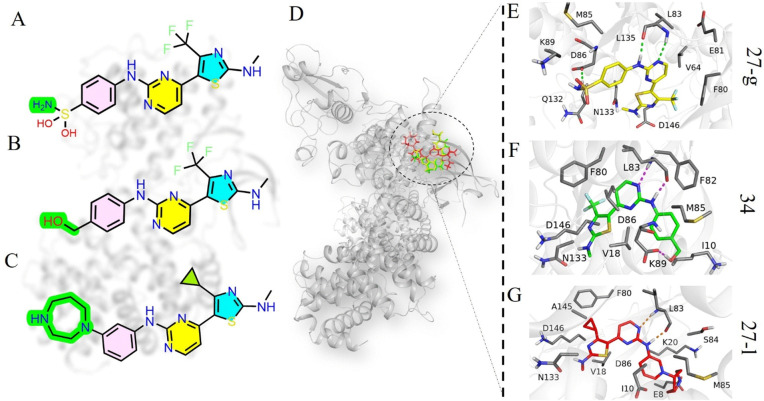
2d Structures of selected compounds(A) 27g (B) 34 (C) 27l Structural binding cavity in CDK1 along with superimposed conformation of 27g, 34, 27l. The binding modes of docked ligands 27g (E), 34 (F), and 27l (G) are illustrated through surrounding pocket residues with their respective binding distances.

To refine our selection, docking scores, binding modes, H-bond interactions, and steric clashes were analyzed, allowing us to identify the highest-scoring ligand-protein complexes. Lower docking scores signified stronger binding affinities, which enabled us to prioritize candidates based on their interactions with pivotal binding pocket residues. Molecular docking, thus, facilitated theoretical predictions of ligand-receptor interactions, particularly useful in the context of inhibitor design. Fig 4A**-**4D showcases the 2D structures and docked poses of three selected ligands within the CDK1 binding site. Among these, compound 27g exhibited high bioactivity with a notable docking score of −12.50, establishing it as a promising candidate. However, compound 34 from our in-house library surpassed 27g, achieving a superior Glide score of −12.61, positioning it as the top inhibitor in this study. Although compound 27l demonstrated a weaker docking score, it was retained for further examination to elucidate its interaction patterns and infer structural features associated with its lower binding affinity. Our results, summarized in Tables S1 and S2 in S1 File in the supporting information, indicate that the in-house compounds yielded more promising results than model compounds, with compounds 34, 27g, and 27l selected for a comparative docking visualization.

Compound 34 demonstrated optimal interactions with critical binding residues, confirming its role as the most potent inhibitor identified. A thorough pocket analysis of compound 34’s docking results, displayed in Fig 4F, reveals its effective coverage of the entire CDK1 binding site, securing interactions with essential residues. Specifically, the hot-spot residue L83 formed dual H-bonds with the central -NH group and N9 atom on the pyrimidine ring, stabilizing the ligand’s binding pose. Additionally, an -OH group attached to the benzene ring exhibited proximity to residue K89, forming a third hydrogen bond that further stabilized the structure. Compound 27g also displayed significant inhibitory potential, validating its effectiveness, while compound 27l, though less active, provided valuable insight into binding poses that do not favor interaction stability. The docked poses of compounds 27g and 27l, depicted in Fig 4E and 4G, reveal differences in their binding modes within the hinge region, particularly with hot-spot residue L83. A minor variation in pose, accompanied by an -NH2 group from the -S(OH)_2_NH_2_ moiety on the benzene ring of 27l, enabled a stable H-bond with residue D86. The divergence in 27 l’s docking pose compared to compounds 34 and 27g may explain its lower activity, as it lacked additional stabilizing interactions. The convergence of both literature-based and newly identified compounds in a consistent docking pose (Fig 4D) underscores this configuration as an optimal binding arrangement for potent CDK1 inhibition. Collectively, these findings highlight the strategic role of molecular docking and virtual screening in pinpointing potential CDK1 inhibitors, setting the stage for the rational design of highly selective compounds that could advance therapeutic development in CDK1-targeted treatments.

### 3.4 MD Simulation

To gain deeper insights into the molecular interactions and stability of the lead compounds, Molecular Dynamics (MD) simulations have been employed. These simulations are pivotal in validating *in-silico* predictions by providing a detailed atomic-level understanding of how these compounds interact with CDK1, thereby offering insights into binding mechanisms, structural stability, and potential modifications for enhanced efficacy. The integration of computational analyses, including molecular dynamics simulations, strengthens the reliability of the predicted ligand-protein interactions and supports the stability of the proposed complexes.

**Fig 5:**
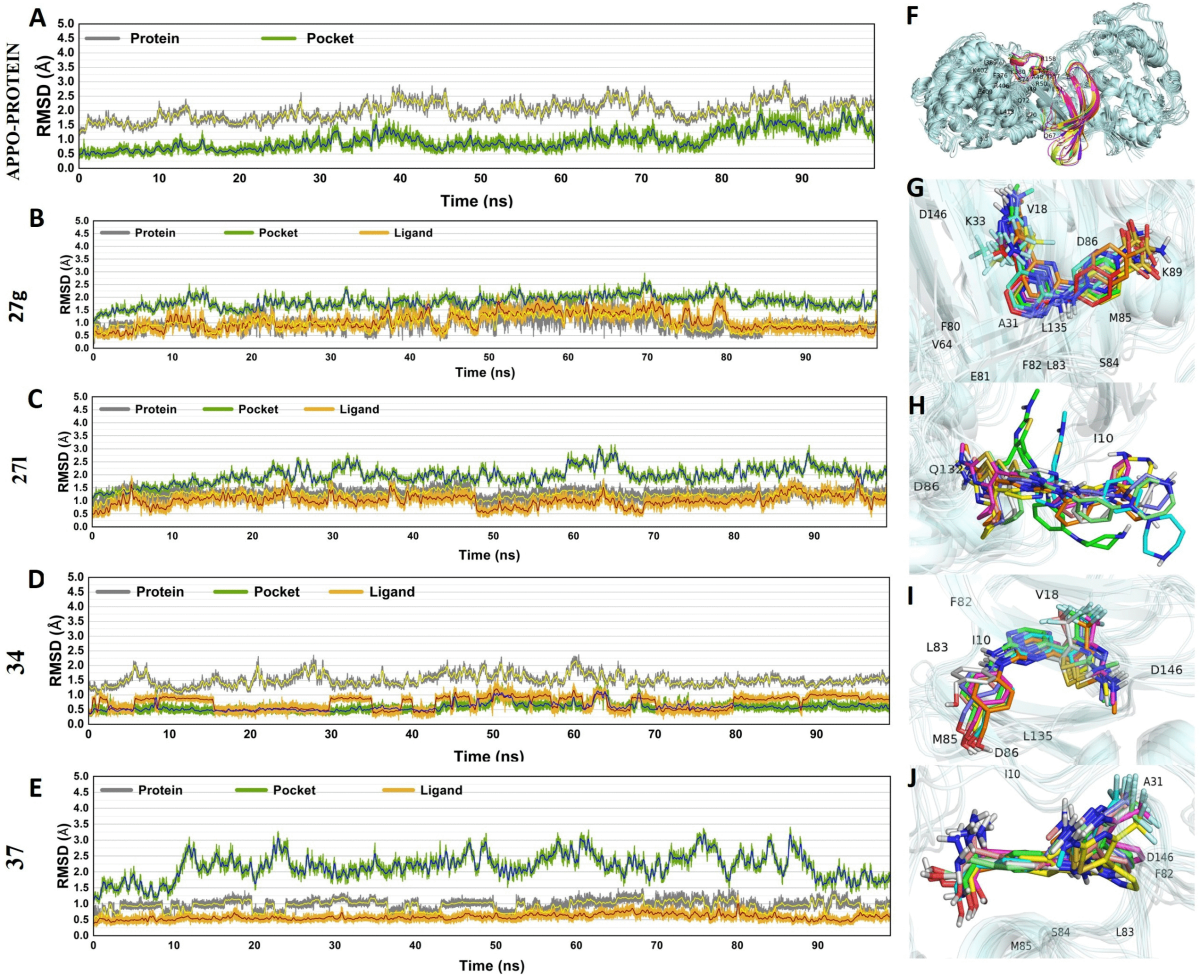
RMSD Analysis and Structural Snapshots of (A) CDK1, 27g(B), 27(C), 34(D), and 37(E) Complexes (Panel 5F-5J) showing MD-simulation snapshot superimposed fluctuations throughout 100 ns, zoom out snapshot shows that the positioning of ligands occupying same binding cavity at particular ns of CDK1, 27g, 27, 34, and 37 complexes respectively.

Molecular dynamics simulations provide a comprehensive computational analysis of atomic-level interactions, including protein dynamics, drug-target engagement, and the complex behavior of ligand-protein complexes. These simulations explored the interaction modes and binding affinities of various inhibitors with CDK1. The dynamic stability of the CDK1 system was assessed over a 100-nanosecond MD simulation by calculating the root-mean-square deviation (RMSD) relative to the selective inhibitors. The RMSD analysis indicated that the system achieved equilibrium within the first 5 nanoseconds. After 30 nanoseconds, the CDK1 protein exhibited minimal fluctuations, indicating a stable equilibrium state. As shown in (Fig 5A), the RMSD values for the protein’s Cα atoms averaged around ∼1.0Å to ∼2.5Å, while the backbone atoms in the binding pocket remained stable with fluctuations of ∼0.5Å to ∼1.5Å, due to residues include L83, V18, D86, F80, A31, and L135 considered as part of the binding pocket are involved in direct interactions with the ligands and contribute to the higher fluctuation observed in the RMSD of the binding pocket compared to the apo protein, which is stable at 1–1.5 Å. (Fig 5B) the RMSD values for the CDK1-27g complex demonstrated the strongest binding affinity and biological activity. The interactions with the protein, binding pocket, and ligand had RMSD values of ∼0.5Å to ∼1.0Å, ∼1.0Å to ∼2.0Å, and ∼0.4Å to ∼1.5Å, respectively. (Fig 5C), shows compound 27l exhibits a slightly higher RMSD due to its binding pose in the hinge region. The higher RMSD reflects some flexibility within the binding pocket, particularly in areas where compound 27l forms fewer stabilizing interactions compared to other potent ligands like compound 34, the RMSD values for both the protein and ligand in the average complex 27l consistently remained between ∼0.5Å and ∼1.0Å throughout the simulation. In contrast, the binding pocket fluctuated around ∼1.0Å to ∼2.0Å, with a peak reaching up to ∼2.5Å between 60 and 70 nanoseconds. This flexibility is likely to contribute to the reduced potency of 27l compared to other compounds

The RMSD analysis (Fig 5D) revealed that the binding pocket and ligand of the best-performing complex 34 remained stable, with values consistently between approximately 0.5Å and 1.0Å, while the protein fluctuated around ~1.0–2.0Å. This indicates stable ligand binding to CDK1 throughout the simulation. In contrast, the CDK1–37 complex (Fig 5E) also exhibited strong binding affinity, with the ligand and protein maintaining RMSD values around ~0.5Å and 1.0Å, respectively. However, the binding pocket showed pronounced fluctuations resembling a rhythmic heartbeat, peaking at 3.0Å, suggesting dynamic pocket behavior during the simulation. (Fig 5F-5J) presents structural overlays and MD snapshots that emphasize the stability of conformations during the simulation. (Fig 5F) The structural superimposition of initial and MD-simulated conformations for the complexes and the APO CDK1 form demonstrates consistent stability throughout the simulation. (Fig 5G-5J) Close-up views of ligand-binding pockets showcase interactions between inhibitors and key residues, highlighting the preservation of hydrogen bonds and hydrophobic interactions: (G) Critical interactions with residues such as D146, K33, V18, and L135 ensure stable ligand binding. (H) The ligands retain contacts with residues like Q132, D86, and I10, underscoring the structural integrity of the binding regions. (I) Stabilizing interactions are maintained with residues A31, E81, F80, and L83, ensuring proximity between ligands and the binding pocket. (J) Hydrophobic contacts involving S84, M85, and L83 remain consistent, further confirming ligand stability within the binding site. This analysis verifies that the MD simulation produced stable complexes, with ligands preserving their initial conformations and essential molecular interactions. These findings provide a robust basis for binding free energy calculations and a deeper understanding of inhibitor interaction mechanisms.

**Fig 6:**
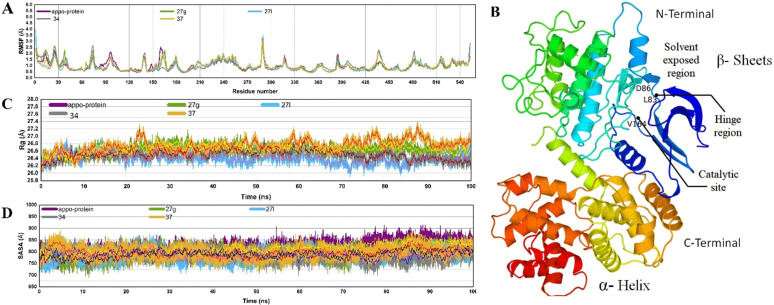
Structural and dynamic behavior of CDK1 in its apo state and complexes with ligands 27g, 27l, 34, and 37. **(A)** highlights RMSF, showing stable hinge and catalytic regions with flexible solvent-exposed areas. **(B)** 3D structure of CDK1, emphasizing key regions. **(C)** Consistent Rg values, indicating compactness, **(D)** SASA variations, reflecting ligand-induced solvent exposure changes.

The Root Mean Square Fluctuation (RMSF) values of key amino acid residues in CDK1, as well as its selected complexes 27g, 27l, 34, and 37, were evaluated to determine residue flexibility (Fig 6A). RMSF values indicate the extent of residue motion, with higher values signifying increased flexibility and lower values indicating rigidity. All ligands displayed similar fluctuation profiles, suggesting a consistent interaction with the protein’s structural dynamics. Notable peaks in flexibility were observed at corresponding residues across all complexes, though the fluctuation magnitudes varied. Specifically, residues around position 290 exhibited significant fluctuations in all complexes, reaching up to ~3.0Å. The apo-CDK1 protein alone displayed a peak of ~2.0Å at residues 95 and 160. Complex 34 showed a peak of ~2.5Å at residue 70, with an additional dominant peak at residue 480. The observed differences in RMSF may indicate variations in binding affinity or complex stability, with higher fluctuations in complexes 37, 34, and 27g suggesting looser binding that permits greater movement, potentially affecting a larger enzyme region. Such variations in residue flexibility could influence CDK1’s catalytic efficiency. Overall, the RMSF analysis indicates that both the apo-protein and all complexes exhibit similar fluctuation patterns, where regions with higher fluctuations imply greater flexibility, which may impact the enzyme’s binding and functional activity. This analysis revealed that the dynamic characteristics and RMSF distributions across the CDK1 protein structure exhibited consistent patterns across all systems. Notable regions within the CDK1 structure the P-loop, α-helix near the hinge region, β-sheets within the catalytic site, the solvent-exposed region, and the hinge region itself displayed distinct fluctuations, as shown in (**Fig 6B**). Specifically, the solvent-exposed region, which includes residues such as D86, exhibited higher flexibility, indicating its dynamic nature during ligand binding. In contrast, the hinge region containing the most crucial residue L83 and the adjacent α-helix remained stable across all systems, underscoring their critical role in maintaining the structural integrity of CDK1 upon ligand association. The β-sheets in the catalytic site exhibited moderate fluctuations, reflecting their role in accommodating ligand binding while maintaining structural stability. These findings highlight the differential flexibility of key structural regions in CDK1 and emphasize the stabilizing influence of the hinge region and α-helix during ligand interactions.

The Radius of Gyration (Rg) analysis (**Fig 6C**) reveals the compactness of the CDK1 protein in both the apo form and its complexes with ligands 27g, 27l, 34, and 37 over a simulation period of 100 ns. Throughout the simulation, all complexes displayed relatively stable Rg values, indicating maintained structural compactness. The apo-protein and complex 27l exhibited the lowest Rg fluctuations, averaging between ~26.0Å to 27.0Å, suggesting a more compact structure. In contrast, complexes 37 and 34 showed slightly higher Rg values, reaching up to ~27.8Å, indicating a relatively less compact structure, which could be attributed to looser binding or conformational changes upon ligand interaction. Complex 27g displayed moderate Rg values, ranging from ~26.4Å to 27.5Å.

The Solvent Accessible Surface Area (SASA) study (**Fig 6D**) determined the protein’s exposure to the solvent. The apo-protein and complex 27l had lower SASA values, varying about ~700–850Å, indicating fewer areas exposed to the solvent and a more compact structure. Complexes 34 and 37 had greater SASA values, peaking at ~950Å, indicating increased surface exposure and potential flexibility or partial unfolding in particular locations. Complex 27g had intermediate SASA values (750–900Å), indicating a moderate amount of exposure. These disparities in SASA indicate that the examined complexes had variable degrees of structural flexibility and stability. Molecular Dynamics simulations highlighted varying interaction dynamics among CDK1 complexes with inhibitors 27g, 27l, 34, and 37. RMSD results showed complexes 27g and 34 with the strongest and most stable binding, while 27l and 37 exhibited moderate flexibility, particularly in their binding pockets. RMSF peaks indicated higher residue fluctuations in 34 and 37, suggesting looser binding. Rg and SASA analyses revealed that the apo-protein and 27l were more compact, whereas 34 and 37 had greater solvent exposure, indicating potential flexibility. Overall, 27g demonstrated the best balance of stability and flexibility, making it the most promising CDK1 inhibitor.

### 3.5. Principal component analysis (PCA) And free energy landscape (FEL) Of Cdk1- along with complexes

**Fig 7:**
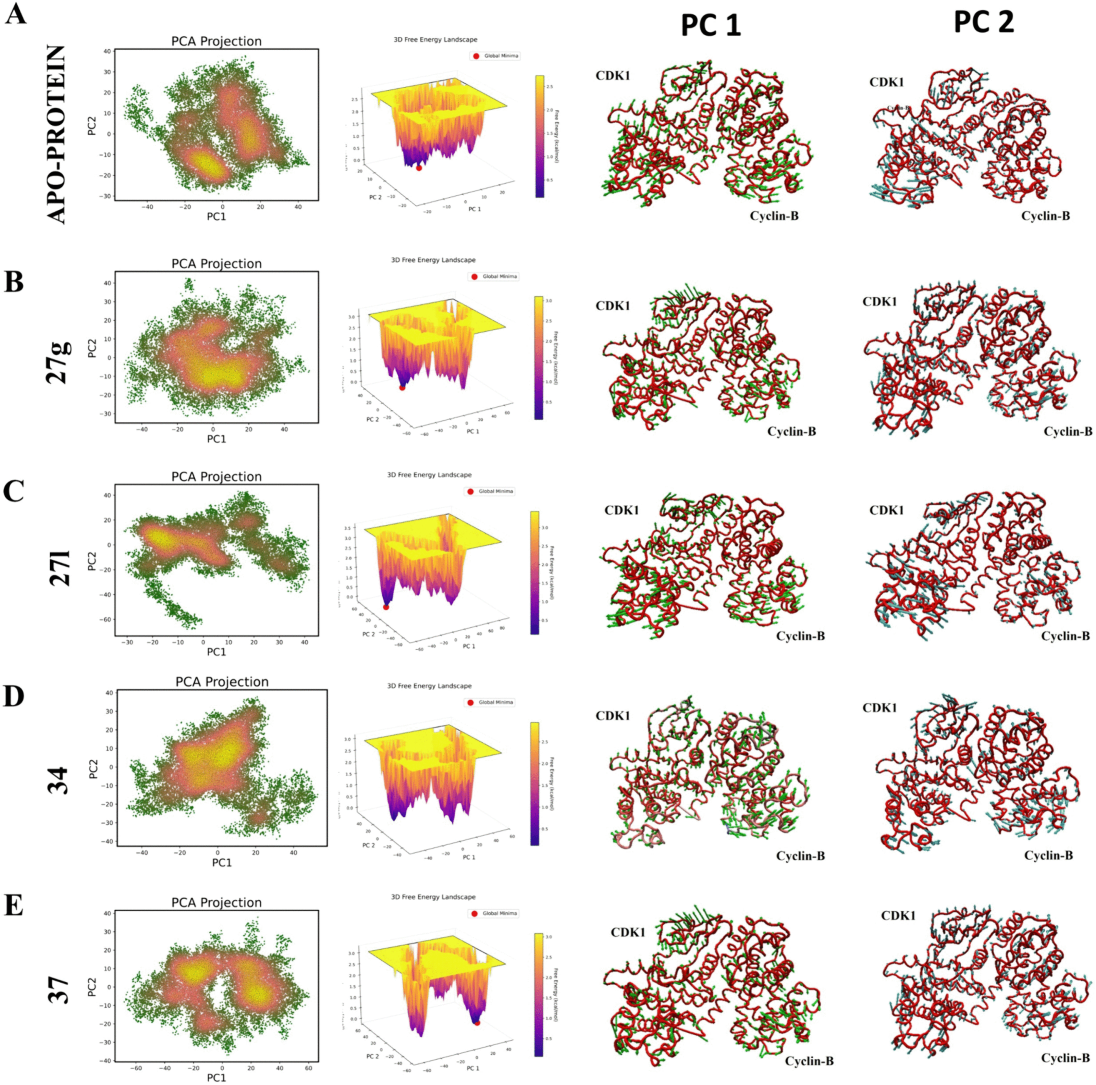
Dynamic behavior of CDK1 in its apo-protein state and complexes with ligands 27g, 27l, 34, and 37 using PCA and Free Energy Landscapes (FEL). PCA projections (left side of Figures A-E) illustrate distinct conformational clustering shown in Yellow and red which indicate stable, low-energy conformations that are consistent with physiologically relevant states of the protein-ligand complex(PCA-Projection), while FELs (center of A-E) display changes in potential energy landscapes. Representative structures in the right-side middle of PC1 and PC2 spaces (A-E) highlight ligand-induced conformational variations, with the 27g and 34 complexes demonstrating stability and the 27l and 37 complexes showing greater conformational diversity.

Principal Component Analysis (PCA) and Free Energy Landscape (FEL) plots were used to examine the dynamic behavior of the CDK1 protein and its ligand-bound complexes, revealing facts about conformational changes and stability during molecular dynamics simulations. The PCA projections of the first two principal components (PC1 and PC2) show significant conformational grouping of CDK1 and its complexes. Each projection shows conformations organized into low-energy basins, denoted by yellow and red patches, indicating stable, physiologically relevant states. The CDK1-APO system has distributed clusters with more structural variation, indicating intrinsic flexibility. The PCA projections of the first two principal components (PC1 and PC2) show significant conformational grouping of CDK1 and its complexes. Each projection shows conformations organized into low-energy basins, denoted by yellow and red patches, indicating stable, physiologically relevant states. The PCA projection of the APO-CDK1 system reveals a scattered distribution of conformations across the PC1 and PC2 axes, reflecting significant intrinsic flexibility in the protein’s structure, with more architectural variation. In contrast, the CDK1-inhibitor complexes demonstrate tighter clustering, suggesting reduced flexibility and enhanced stabilization upon ligand binding. Among the analyzed complexes, CDK1-27g demonstrates the most stable conformations. The PCA projection shows a tightly clustered distribution in the low-energy basin, suggesting minimal conformational diversity. The CDK1-27l complex exhibits moderate stability, as reflected in its PCA projection, where conformations are more dispersed than CDK1-27g but less scattered than APO-CDK1. The CDK1–34 complex stabilizes significantly, as evidenced by its PCA projection, where conformations are densely clustered within a well-defined low-energy basin. The CDK1–37 complex ranks as the third-best in terms of stability. Its PCA projection shows clustering similar to that of CDK1–34 but with slightly more dispersed conformations, shown on the left side of (Fig 7A-7E). The PCA projections reveal unique dynamics and stability among CDK1 systems. APO-CDK1 has the most flexibility, whereas CDK1-27g is extremely stable with tight clustering in low-energy basins. CDK1-27l has moderate stability, CDK1–34 exhibits high stability with minor dispersion, and CDK1–37 exhibits intermediate stability, ranking behind CDK1–34. These results highlight the stabilizing impact of particular ligands on CDK1 conformations.

#### 3.5.1. Free energy landscape (FEL) Of Cdk1- along with complexes.

The FEL overlays on the PCA projections in Fig 7 offer a quantitative view of energy minima, providing insights into the stability of CDK1 systems. For CDK1-APO, the global minimum is located within a broad, shallow basin, reflecting less constrained energy states and significant conformational variability, with energy values around −22.5 kcal/mol. This suggests a highly flexible and dynamic structural landscape for the unbound protein. The FEL overlays reveal distinct energy landscapes for CDK1 systems, highlighting the stabilizing effects of ligand binding. CDK1-ligand complexes exhibit deeper energy wells than unbound CDK1, indicating energetically favorable states and conformational stabilization. The FEL plot for CDK1-27g displays the deepest global minimum at approximately −35.7 kcal/mol, reflecting the highest stability among the complexes. Despite the similar PCA projections of compounds 27l and 34, their free energy landscapes differ due to variations in binding interactions. Compound 34 forms additional stabilizing hydrogen bonds, leading to a more favorable free energy landscape. In contrast, compound 27l lacks these stabilizing interactions, contributing to a less favorable energy profile. CDK1–34 follows closely with a worldwide minimum of −33.8 kcal/mol, suggesting significant stabilization. CDK1-27l shows a moderately deep energy minimum at −30.2 kcal/mol, indicating partial stabilization, while CDK1–37 presents a minimum at −32.5 kcal/mol, representing a stable yet slightly less favorable state than CDK1-27g and CDK1–34.

The FEL analysis reveals distinct stability profiles for CDK1 systems, with APO-CDK1 displaying the greatest flexibility and the ligand-bound complexes showing enhanced stabilization. CDK1-27g exhibits the highest stability, followed by CDK1–34 and CDK1–37, while CDK1-27l demonstrates moderate stabilization. These findings emphasize the ligand-induced conformational locking of CDK1 into low-energy, stable states, contrasting with the broader, shallow minimum of unbound CDK1 (−22.5 kcal/mol), which reflects greater flexibility and dynamic variability.

#### 3.5.2. Porcupine plots of atom motion.

The porcupine plots in Fig 7 (rightmost columns) provide a visual representation of atomic fluctuations, with arrows denoting the magnitude and direction of Cα atom movements, In APO-CDK1, prominent fluctuations are observed in the hinge region, solvent-exposed loops, and β-sheet domains, underscoring the protein’s inherent flexibility. This dynamic nature enables APO-CDK1 to explore diverse conformational states, facilitating potential ligand interactions.

The porcupine plot for CDK1-27g shows minimal atomic fluctuations in the catalytic site and hinge region, highlighting the ligand’s ability to stabilize key binding residues such as D86 and L83. This stability positions CDK1-27g as the most effective complex in terms of inhibitory potential. In CDK1-27l, slight flexibility is observed in the solvent-exposed loops and β-sheets, while the catalytic and hinge regions remain relatively stable, suggesting a balance between flexibility and stability that supports functionality but is less optimal. For CDK1–34, atomic motion is largely confined to distal regions, with the catalytic site and hinge region exhibiting rigid structural integrity, marking it as the second-best complex. The porcupine plot for CDK1–37 reveals moderate fluctuations in the β-sheets and solvent-exposed regions, while the catalytic and hinge regions remain stable. This indicates good stabilization, though slightly less efficient than CDK1–34.

The PCA and FEL analyses collectively underscore the dynamic interplay between stability and flexibility in CDK1 and its complexes. Ligand binding stabilizes the hinge region and key secondary structures, such as β-sheets and α-helices, while allowing minor flexibility in solvent-exposed loops and distal regions. These structural adjustments enable optimal ligand accommodation while preventing unfavorable conformational shifts that could activate CDK1. Notably, the observed minima for CDK1-27g suggest an exceptional binding affinity, making it a promising candidate for further optimization in therapeutic design.

These findings highlight the conformational preferences and dynamic adaptability of CDK1, offering valuable insights for designing potent inhibitors targeting its active site. Future investigations focusing on flexible regions identified in these analyses could further enhance the inhibitory potential of CDK1-targeted therapies.

### 3.6. Cross correlation analysis

**Fig 8:**
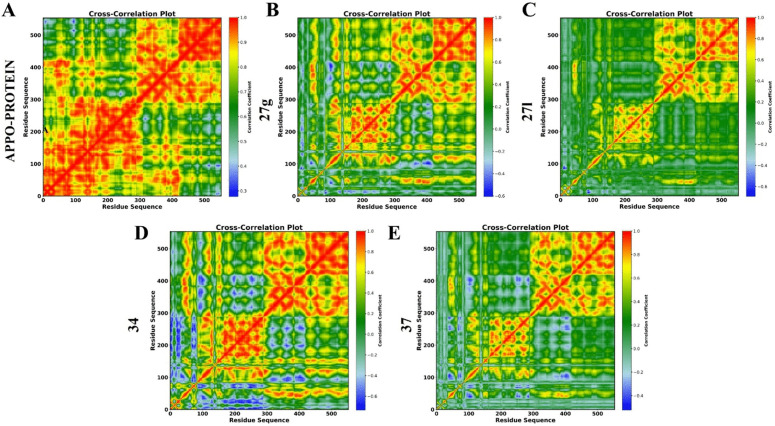
Cross-Correlation Analysis of (A) CDK1, along with its complexes 27g(B), 27l(C), 34(D), and 37(E); revealing the correlated motions between different residues within each complex.

Dynamic cross-correlation maps (DCCM) were used to understand the residual motion within the protein system. This relation is determined through positive and negative correlation among residues. Positive values signal the correlated threshold of residues while negative values indicate the anti-correlation over the period of MD simulation. The pink color indicates a positive correlation while green color depicts negative correlation. The cross-correlation plot revealed significant interactions between key residues in CDK1. Notably, the residues in the hinge region (F10, L11, and M12) exhibited strong positive correlations, suggesting concerted movements within this region. Additionally, residues in the shallow hydrophobic cavity (I20 and V21) showed a high degree of correlation with each other and with the hinge region residues (F10, L11, and M12), indicating a potential role in ligand binding and conformational changes. The catalytic region residues (K30 and E31) also demonstrated significant correlations with both the hinge and cavity residues (F10, L11, M12, I20, and V21), highlighting their involvement in the overall dynamics of the protein. These findings suggest that the hinge region, shallow hydrophobic cavity, and catalytic region work in concert to facilitate ligand binding and subsequent enzymatic activity in CDK1 as shown in Fig 8A.

DCCM were employed to investigate the dynamic motions within the CDK1-27g complex, (Fig 8B) a complex selected for further study due to the promising potential for interaction, as indicated by high docking scores of compound 27g. This analysis revealed intricate correlations among residues, providing insights into their collective movements. A closer examination of the DCCM highlights several key observations. Residues within the hinge region (F10, L11, and M12) exhibit pronounced positive correlations, suggesting concerted movements within this crucial structural element. Furthermore, residues residing in the shallow hydrophobic cavity (I20 and V21) demonstrate a high degree of correlation with each other and with the hinge region residues, suggesting a concerted role in ligand binding and subsequent conformational changes. Importantly, the catalytic region residues (K30 and E31) also display significant correlations with both the hinge and cavity residues. This intricate network of correlated motions underscores the dynamic interplay between these regions, emphasizing their collective contribution to the overall function of the CDK1-27g complex.

CDK1-27l complex exhibits unique features in its cross-correlation map as shown in (Fig 8C) Notably, a more pronounced diagonal band is observed, suggesting heightened correlations between residues along the protein sequence. This may indicate more extensive concerted domain motions within the CDK1-27l complex than other complexes. Furthermore, the localized regions of high correlation, particularly those involving residues directly interacting with compound 27l (F10, L11, M12, I20, V21, K30, and E31), appear more distinct, suggesting a more tightly coupled network of interactions within this complex.

DCCM revealed intricate residue correlations within the CDK1–34 complex. Hinge region residues (F10, L11, M12) exhibited strong positive correlations, suggesting concerted movements. Residues in the hydrophobic cavity (I20, V21) displayed a high correlation with both the hinge and each other, while catalytic region residues (K30, E31) showed significant correlations with both. This network of correlated motions, particularly involving ligand-interacting residues, underscores their collective contribution to the complex’s function. The DCCM exhibited a prominent diagonal band, indicative of extensive concerted domain motions, and localized regions of high correlation, suggesting functionally important sites. Notably, the CDK1–34 complex displayed a more pronounced diagonal band and distinct localized regions as illustrated in (Fig 8D) compared to CDK1 alone, suggesting a more tightly coupled network of interactions within this complex, DCCM revealed intricate residue correlations within the CDK1–37 complex. Hinge region residues (F10, L11, M12) exhibited pronounced positive correlations, suggesting concerted movements. Residues in the hydrophobic cavity (I20, V21) and catalytic region (K30, E31) showed significant correlations with each other and with the hinge. The cross-correlation plot displayed a prominent diagonal band, indicative of concerted domain motions (Fig 8E). Notably, the CDK1–37 complex exhibited a more pronounced diagonal band and distinct localized regions compared to previous analyses, suggesting a more tightly coupled network of interactions.

DCCM revealed intricate interactions within the CDK1 protein system across different complexes. Consistent across all complexes, the hinge, hydrophobic cavity, and catalytic regions showed significant positive correlations, highlighting their functional importance. The CDK1-27l and CDK1–34 complexes exhibited broader concerted domain motions and tighter interaction networks. However, the CDK1–37 complex demonstrated the most prominent diagonal band and distinct localized regions of high correlation in its DCCM, suggesting the most tightly coupled network of interactions and potentially superior ligand binding and enzymatic activity. Further studies are necessary to validate these findings and determine the most promising candidate.

**Fig 9:**
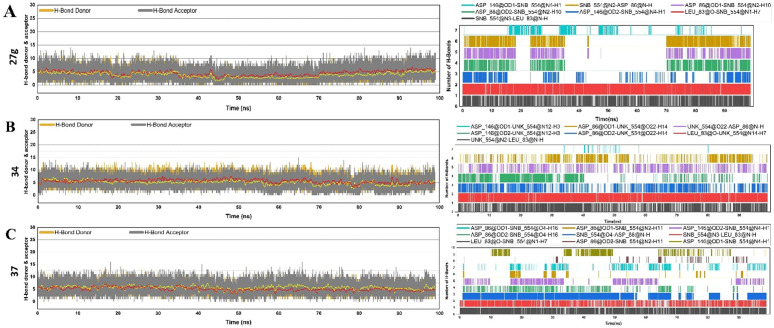
Hydrogen bonding patterns in CDK1-27g, CDK1-34, and CDK1-37 complexes. The left side of Figures (A-C) illustrates the time evolution of hydrogen bond donors and acceptors throughout the simulation, showcasing their stability and frequency. The right side of Figures (A-C) depicts hydrogen bond timelines, pinpointing specific residues involved in consistent interactions during the trajectory, emphasizing the importance of hydrogen bonds in stabilizing ligand-bound CDK1 complexes.

Fig 9 illustrates the hydrogen bond dynamics of the CDK1-ligand complexes throughout the MD simulations. The CDK1-27g complex maintained a relatively stable hydrogen bond network, with an average of approximately 14–16 hydrogen bonds observed over the simulation period, indicating persistent ligand engagement within the binding pocket (Fig 9A). In comparison, the CDK1–34 complex exhibited a moderately higher level of hydrogen bond interactions, maintaining around 18–20 hydrogen bonds, reflecting a more flexible yet stable interaction pattern during the simulation (Fig 9B). Notably, the CDK1–37 complex displayed the most dynamic hydrogen bond behavior, with a comparatively higher hydrogen bond count of approximately 22–25, accompanied by frequent rearrangements, suggesting enhanced conformational adaptability within the binding site (Fig 9C). Overall, this comparative analysis demonstrates that hydrogen bonding contributes differently to the stability and dynamic behavior of each complex, thereby influencing their interaction profiles and functional characteristics. For completeness, the hydrogen bond interaction pattern of the comparatively weaker ligand CDK1-27l, which exhibited limited and transient hydrogen bonding during the simulation, is provided in **Fig S3 in** S1 File.

### 3.9. Binding energy analysis

The binding free energy analysis for the complexes (27g, 27l, 34, and 37) was conducted using molecular mechanics Poisson–Boltzmann surface area (MM/PBSA)

molecular mechanics generalized Born surface area *(MM/GBSA)* methods, incorporating key energy components. Complex 37 demonstrated the strongest van der Waals interactions with a ΔE_vdw_ of −46.14 kcal/mol, followed by 27l, 27g, and 34, with values of −45.45 kcal/mol, −44.50 kcal/mol, and −44.32 kcal/mol, respectively. Electrostatic interactions (ΔE_ele_) were most significant in 27g (−50.49 kcal/mol) and 37 (−44.22 kcal/mol), indicating stronger charge-based binding, whereas 27l had the weakest electrostatic component (−16.76 kcal/mol), highlighting a distinct binding mechanism.

**Fig 10:**
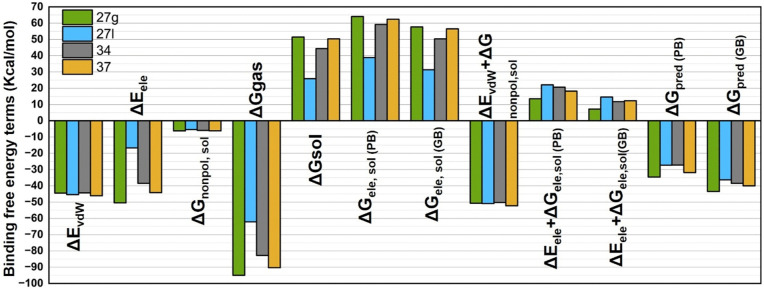
The binding free energy components calculated using Molecular Mechanics Poisson-Boltzmann Surface Area (MMPBSA) Analysis for CDK1 and its ligands; 27g, 27l, 34, and 37.

The gas-phase free energy (Δg_gas_), a combination of ΔE_vdw_ and ΔE_ele_, was highest for complex 27g (−94.99 kcal/mol), indicating superior binding affinity without solvent interference, closely followed by complex 37 (−90.36 kcal/mol). Complex 34 and 27l displayed Δg_gas_ values of −82.87 kcal/mol and −62.21 kcal/mol, respectively, suggesting varying degrees of interaction strength. Nonpolar solvation energy (ΔG_nonpol_, _sol_) values were relatively consistent across complexes, with 27g and 37 having slightly lower values (−6.21 and −6.17 kcal/mol, respectively), indicating enhanced stability in a hydrophobic environment, while 27l had the highest value (−5.45 kcal/mol), suggesting slightly less hydrophobic stabilization as shown in Fig 10. To conclude, complex 27g exhibits the strongest binding affinity, primarily driven by its robust electrostatic interactions, making it the most stable in the absence of solvent. Complex 37 closely follows with strong van der Waals and electrostatic interactions, showing competitive binding strength. Complex 34 demonstrates moderate binding, while 27l ranks the lowest due to its weaker electrostatic interactions and higher solvation energy, resulting in reduced stability. Thus, 27g is the most promising compound, followed by 37, with 34 and 27l being less effective.

### 3.10. ADME analysis

The ADME analysis of the CDK1-targeted compounds from the in-house library, provided as **Table S3 in** S1 File in the supporting information, includes pharmacokinetic parameters, synthetic accessibility, and toxicity profiles. The table highlights critical factors, such as intestinal absorption, volume of distribution (VDss), cytochrome P450 interactions, total clearance rates, and AMES toxicity predictions. Most compounds demonstrated high intestinal absorption, a key indicator of favorable oral bioavailability, and adhered to Lipinski’s rule of five with minimal violations, supporting their drug-likeness. Synthetic accessibility scores ranged between 3.3 and 4.0 for the majority of compounds, indicating ease of synthesis. AMES toxicity tests suggested a predominantly non-mutagenic and non-carcinogenic profile across the library. Among these, compounds 34 and 37 emerged as exceptional candidates. Compound 34 displayed an absorption rate of 88.633%, coupled with a balanced distribution profile (VDss: −0.062 log L/Kg) and a synthetic accessibility score of 3.82, confirming its feasibility for practical synthesis. Its inhibition of multiple CYP450 isoforms, including CYP1A2, CYP2C9, and CYP3A4, points to its robust metabolic stability, although it necessitates caution for potential drug-drug interactions. Compound 37, with an absorption rate of 70.091% and a VDss of 0.944 log L/Kg, showed slightly higher synthetic accessibility (3.99) but retained its drug-likeness features. It selectively inhibited CYP3A4, minimizing risks associated with extensive CYP450 involvement. Both compounds were non-carcinogenic and non-mutagenic, further enhancing their profiles as safe and effective candidates for CDK1 inhibition. These findings underscore compounds 34 and 37 as promising leads, warranting further investigation and development in the context of CDK1-targeted therapies.

## 4. Conclusion

This study presents a comprehensive computational investigation into the design of potent CDK1 inhibitors, leveraging 3D-QSAR modeling, molecular docking, and MD simulations. The analysis identified key structural features and interaction patterns critical for robust binding to CDK1, emphasizing the significance of residues such as L83 and V18. Compounds 34 and 37 emerged as promising candidates, demonstrating high docking scores, stable MD simulation results, and favorable ADMET profiles. These findings highlight the potential of computational approaches in accelerating the discovery of CDK1 inhibitors with high specificity and efficacy. Furthermore, the insights gained from this study into the binding dynamics and pharmacokinetics of CDK1 inhibitors offer a strong foundation for future experimental validation and optimization. By enhancing binding interactions and minimizing off-target effects, these compounds promise to advance oncology therapeutic interventions. Expanding the structural diversity of the inhibitor library and integrating experimental approaches will be essential next steps in translating these computational findings into clinically viable treatments.

## Supporting information

S1 File Supplementary figures, tables, and computational data supporting the molecular modelling, 3D-QSAR, docking, and molecular dynamics studies of pyrazolopyrimidine-based CDK1 inhibitors. (DOCX)
