# Supplementary material for: Molecular modeling of highly selective CDK1 Inhibitors based on pyrazolo-pyrimidines using 3D-QSAR, docking, and molecular dynamics simulations
Source: PLoS One. 2026 Jun 22;21(6):e0350566. doi: 10.1371/journal.pone.0350566 (PMC13286157; doi:10.1371/journal.pone.0350566)
Supplement: S1 File — (DOCX) [file pone.0350566.s001.docx]

**Molecular modelling of pyrazolopyrimidine-based CDK1 inhibitors integrating 3D-QSAR, molecular docking, and molecular dynamics simulations**

Muhammad Afaq Tahir^1,2^, Tahir Ali Chohan^1^*, Aisha qayyum^3^**, Abdullah Yahya Abdullah Alzahrani^4^, Abdullah R. Alzahrani^5^, Zia Ur Rehman^6,7^, Abida Khan^8^, Khuram Ashfaq^9^

*^1^Institute of Pharmaceutical Sciences, university of veterinary and Animal Sciences, Lahore, 54000, Pakistan.*

*^2^Department of Pharmacy, Faculty of Pharmaceutical Sciences, Green International University, Lahore-54900, Pakistan.*

*^3^Department peadiatric medicine, Fatima memorial hospital, Lahore, Pakistan*

*^4^Department of Chemistry, Faculty of Science, King Khalid University, Abha, Kingdom of Saudi Arabia.*

*^5^Department of Pharmacology and Toxicology, Faculty of Medicine, Umm Al-Qura University, Al-Abidiyah, P.O.Box 13578, Makkah, 21955, Saudi Arabia.*

*^6^Health Research Centre, Jazan University, P.O. Box 114, Jazan 45142, Saudi Arabia. ^7^Department of Pharmaceutical Chemistry, Faculty of Pharmacy, Jazan University, P.O. Box 114 (Postal Code: 45142), Jazan, Kingdom of Saudi Arabia.*

*^8^Center For Health Research, Northern Border University, Arar 73213, Saudi Arabia.*

*^9^Faculty of Pharmaceutical sciences, Lahore University of biological and Applied Sciences.*

*Correspondence: [tahir.chohan@uvas.edu.pk](mailto:tahir.chohan@uvas.edu.pk),

[aisha.qayyum86@gmail.com](mailto:aisha.qayyum86@gmail.com),


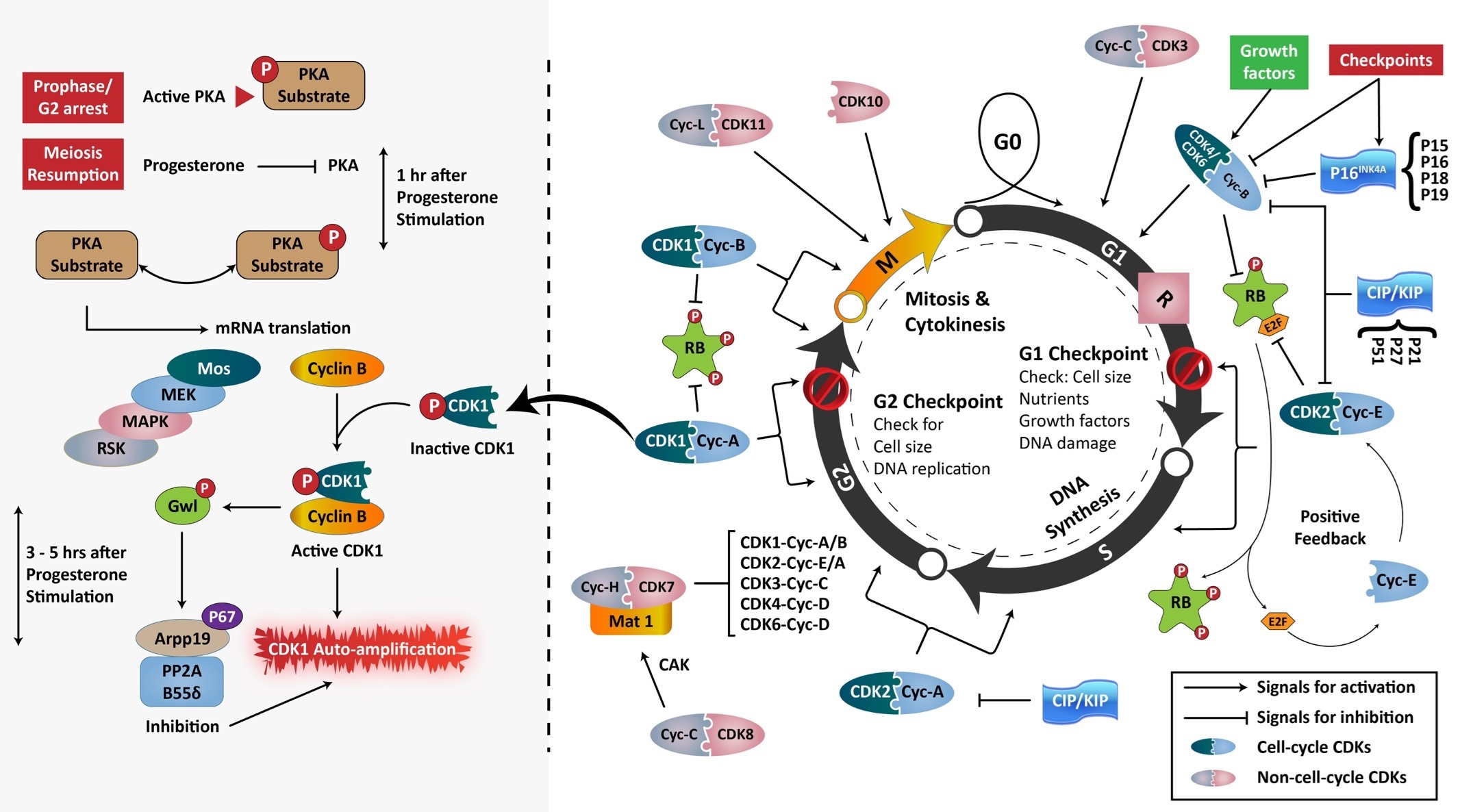


**Fig S1:** Schematic representation of the mechanistic role of CDK1 in cell cycle regulation, illustrating its interaction with cyclins, checkpoint control, regulatory phosphorylation events, and involvement in G2/M transition and mitotic progression. The diagram summarizes key regulatory pathways governing CDK1 activation and inhibition, as previously reported by the authors.

**
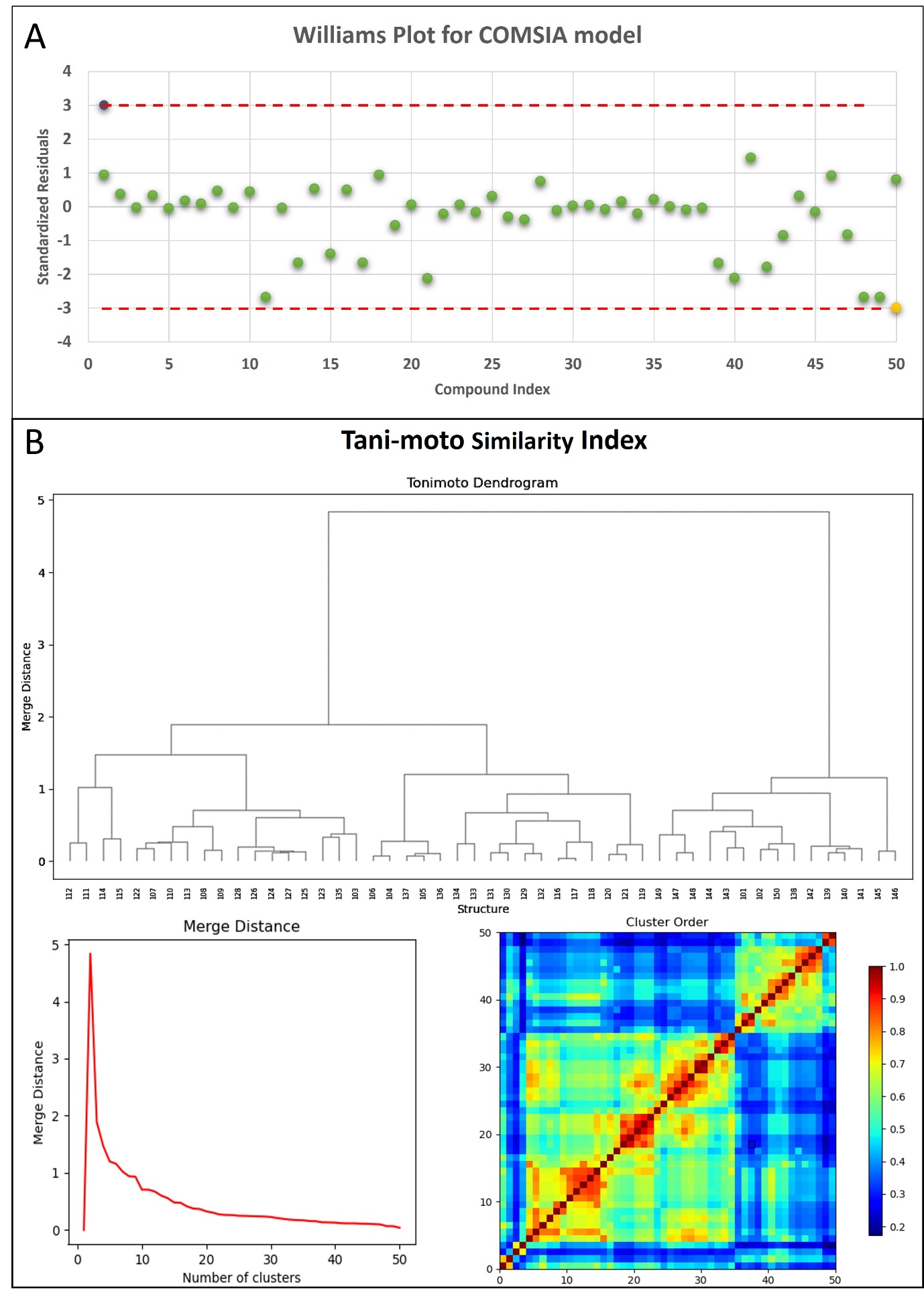
**

**Fig S2:** Applicability domain analysis of the CoMSIA model. (A) Williams plot of standardized residuals showing that most compounds lie within the acceptable limits (±3). (B) Tanimoto similarity-based hierarchical clustering demonstrating that the compounds are well distributed within the defined chemical space.

**
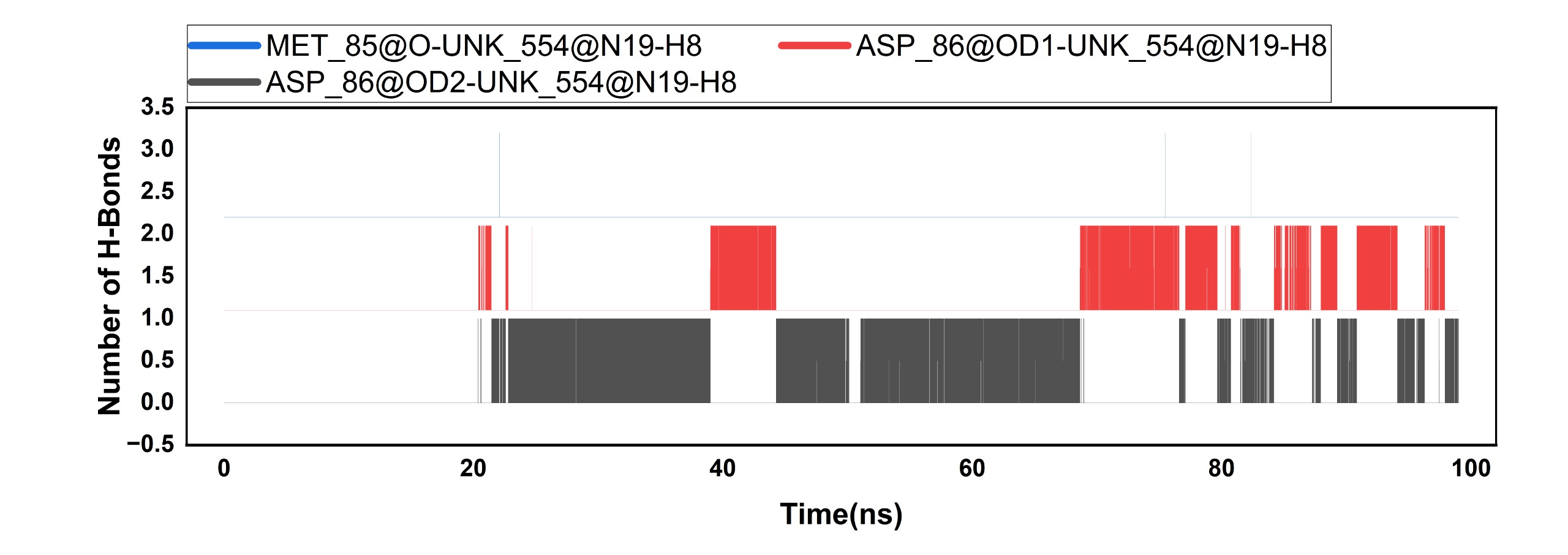
**

**Fig S3:** Hydrogen bond interaction profile of the CDK1–27l complex during the 100 ns molecular dynamics simulation. The plot illustrates the time-dependent formation of hydrogen bonds between compound 27l and key CDK1 residues, highlighting the limited and transient nature of hydrogen bond interactions compared to the other investigated complexes.

**Table S1: Structures, experimental and predicted inhibitory activities of the 3D-QSAR modeling data sets.**

|  |
| --- |
| \| **Compound name** \| **R_1_** \| **R_2_** \| **R_3_** \| **R_4_** \| **R_5_** \| **IC_50_ (nΜ)** \| **pIC_50_** \| **CoMFA** \| \| **CoMSIA** \| \| **Docking Score** \| \| --- \| --- \| --- \| --- \| --- \| --- \| --- \| --- \| --- \| --- \| --- \| --- \| --- \| \| **Training set** \| \| \| \| \| \| \| \| \| \| \| \| \| \|  \|  \|  \|  \|  \|  \|  \|  \| **Pred** \| **Res** \| **Pred** \| **Res** \|  \| \| 1a \| H \| Me \| H \|  \| - \| 5 \| 8.301 \| 8.105 \| 0.196 \| 7.82 \| 0.481 \| -9.292 \| \| 1b \| H \| Me \| H \|  \| - \| 6 \| 8.222 \| 8.411 \| -0.189 \| 8.03 \| 0.192 \| -9.55 \| \| 9a \| F \| Me \| H \|  \| - \| 7 \| 8.155 \| 8.147 \| 0.008 \| 8.17 \| -0.015 \| -10.451 \| \| 9b \| F \| Me \| H \|  \| - \| 41 \| 7.387 \| 7.343 \| 0.044 \| 7.22 \| 0.167 \| -6.383 \| \| 9c \| F \| Me \| Me \|  \| - \| 32 \| 7.495 \| 7.348 \| 0.147 \| 7.52 \| -0.025 \| -5.903 \| \| 9d \| F \| Me \| H \|  \| - \| 65 \| 7.187 \| 7.306 \| -0.119 \| 7.1 \| 0.087 \| -7.905 \| \| 9e \|  \| Me \| Me \|  \| - \| 19 \| 7.721 \| 7.742 \| -0.021 \| 7.68 \| 0.041 \| -8.861 \| \| 9f \|  \| Me \| Me \|  \| - \| 34 \| 7.469 \| 7.199 \| 0.27 \| 7.23 \| 0.239 \| -5.336 \| \| 9j \| F \| Me \| Me \|  \| Me \| 101 \| 6.996 \| 6.819 \| 0.177 \| 7.01 \| -0.014 \| -7.336 \| \| 9k \|  \| Me \| Me \|  \| - \| 576 \| 6.24 \| 6.25 \| -0.01 \| 6.01 \| 0.23 \| -5.163 \| \| 9m \|  \| Me \| Me \|  \| - \| 205 \| 6.58 \| 6.899 \| -0.319 \| 7.96 \| -1.38 \| -5.433 \| \| 9n \|  \| Me \| Me \|  \| - \| 166 \| 6.78 \| 6.357 \| 0.423 \| 6.8 \| -0.02 \| -7.509 \| \| 9p \|  \| Me \| Me \|  \| Cl \| 5000 \| 5.301 \| 5.658 \| -0.357 \| 6.158 \| -0.857 \| -6.232 \| \| 9q \| F \| Me \| Me \|  \| - \| 64 \| 7.194 \| 7.221 \| -0.027 \| 6.921 \| 0.273 \| -8.494 \| \| 9r \|  \| Me \| Me \|  \| - \| 43 \| 7.367 \| 7.487 \| -0.12 \| 8.087 \| -0.72 \| -8.402 \| \| 12a \|  \| Me \| Me \|  \| - \| 6 \| 8.222 \| 8.585 \| -0.363 \| 7.97 \| 0.252 \| -8.646 \| \| 12d \| OH \| Me \| Me \|  \| - \| 5000 \| 5.301 \| 5.658 \| -0.357 \| 6.158 \| -0.857 \| -9.969 \| \| 12e \| F \| Me \| Me \|  \| - \| 4 \| 8.301 \| 8.105 \| 0.196 \| 7.82 \| 0.481 \| -9.407 \| \| 12f \| Cl \| Me \| Me \|  \| - \| 19 \| 7.721 \| 7.271 \| 0.45 \| 8.01 \| -0.289 \| -9.618 \| \| 12g \| Me \| Me \| Me \|  \| - \| 62 \| 7.208 \| 7.174 \| 0.034 \| 7.18 \| 0.028 \| -9.163 \| \| 12h \| Et \| Me \| Me \|  \| - \| 788 \| 6.103 \| 6.636 \| -0.533 \| 7.2 \| -1.097 \| -6.005 \| \| 12k \|  \| Me \| Me \| - \|  \| 42 \| 7.377 \| 7.34 \| 0.037 \| 7.49 \| -0.113 \| -9.142 \| \| 12l \|  \| Me \| Me \| - \|  \| 45 \| 7.347 \| 7.184 \| 0.163 \| 7.32 \| 0.027 \| -9.618 \| \| 12m \|  \| Me \| Me \|  \| - \| 79 \| 7.102 \| 7.189 \| -0.087 \| 7.19 \| -0.088 \| -6.64 \| \| 12n \|  \| Me \| Me \| 6- \| - \| 35 \| 7.456 \| 7.61 \| -0.154 \| 7.3 \| 0.156 \| -6.027 \| \| 12o \|  \| Me \| Me \| 6- \| - \| 26 \| 7.585 \| 7.777 \| -0.192 \| 7.74 \| -0.155 \| -5.842 \| \| 12p \| F \| Me \| Me \|  \| - \| 24 \| 7.62 \| 7.553 \| 0.067 \| 7.82 \| -0.2 \| -6.734 \| \| 12q \| C \| Me \| Me \| 1- \| - \| 18 \| 7.745 \| 7.362 \| 0.383 \| 7.36 \| 0.385 \| -9.896 \| \| 12s \| F \| Me \| Me \| 1- \| - \| 47 \| 7.328 \| 7.473 \| -0.145 \| 7.39 \| -0.062 \| -8.549 \| \| 27a \| H \| Me \| Me \| 1- \| - \| 5000 \| 5.301 \| 5.139 \| 0.162 \| 5.29 \| 0.011 \| -9.291 \| \| 27b \| H \|  \| Me \| 1- \| - \| 5000 \| 5.301 \| 5.737 \| -0.436 \| 5.28 \| 0.021 \| -6.405 \| \| 27d \| H \|  \| Me \| - \| -OH \| 334 \| 6.476 \| 6.403 \| 0.073 \| 6.52 \| -0.044 \| -5.506 \| \| 27f \| H \|  \| Me \|  \| - \| 1.5 \| 8.824 \| 8.95 \| -0.126 \| 8.75 \| 0.074 \| -10.423 \| \| 27g \| H \|  \| Me \| - \|  \| 0.5 \| 9.301 \| 9.153 \| 0.148 \| 9.41 \| -0.109 \| -12.50 \| \| 27h \| H \| H \| Me \| - \|  \| 1 \| 9 \| 9.013 \| -0.013 \| 8.89 \| 0.11 \| -9.111 \| \| 27i \| H \| H \| Me \|  \| - \| 4 \| 8.398 \| 8.577 \| -0.179 \| 8.4 \| -0.002 \| -10.097 \| \| 27k \| H \|  \| Me \| _-_ \|  \| 2 \| 8.699 \| 8.562 \| 0.137 \| 8.75 \| -0.051 \| -8.151 \| \| 27l \| H \|  \| Me \| 1- \| - \| 176 \| 6.58 \| 6.347 \| 0.453 \| 7.0 \| -0.02 \| -4.625 \| |

**Test Compounds**

|  | **R_1_** | **R_2_** | **R_3_** | **R_4_** | **IC_50_ (nM)** | **pIC_50_** | **CoMFA** | | | **CoMSIA** | | **Docking Score** |
| --- | --- | --- | --- | --- | --- | --- | --- | --- | --- | --- | --- | --- |
| **Test set** | | | | | | | | | | | | |
|  |  |  |  |  |  |  | **Pred** | | **Res** | **Pred** | **Res** |  |
| 1c | H | Me | Me | H | 195 | 6.71 | 7.469 | | -0.759 | 7.572 | -0.862 | -8.934 |
| 12b | OH | Me | Me | 1- | 1424 | 5.84 | 7.93 | | -2.09 | 6.93 | -1.09 | -8.803 |
| 12j |  | Me | Me | 1- | 43 | 7.36 | 7.214 | | 0.146 | 6.62 | 0.74 | -6.207 |
| 27c | H |  | Me | 1-OH | 556 | 6.25 | 6.402 | | -0.152 | 7.17 | -0.92 | -5.875 |
| 27e | H |  | Me | 1- | 154 | 6.81 | 6.516 | | 0.294 | 7.25 | -0.44 | -5.476 |
| 27j | H |  | Me | 1- | 18 | 7.74 | 7.706 | 0.034 | | 7.58 | 0.16 | -7.693 |
| 9i | H | Me | Me | 1- | 357 | 6.447 | 7.1 | -0.653 | | 6.53 | -0.083 | -8.549 |
| 9g | H | Me | Me | 1- | 10 | 8 | 7.47 | 0.53 | | 7.53 | 0.47 | -8.661 |
| 9o |  | Me | Me | 1- | 313 | 6.5 | 6.015 | 0.485 | | 6.93 | -0.43 | -7.237 |
| 9l | H | Me | Me | 1- | 261 | s | 6.91 | -0.31 | | 7.98 | -1.38 | -8.908 |
| 9s |  | Me | Me | 1- | 262 | 6.58 | 6.899 | -0.319 | | 7.96 | -1.38 | -7.908 |
| 9h |  | Me | Me | 1- | 73 | 7.137 | 6.928 | 0.209 | | 6.725 | 0.412 | -8.342 |

IC_50_: Half-maximal inhibitory concentration, Pred: Predicted IC_50_ values, pIC_50_: Negative log of IC_50_ Res: Difference between actual and predicted IC_50_ values, Docking score _=_Glide docking score.

**Table S2:**  **Predicted inhibitory activities compound and docking scores of in-house library.**

|  | **LIGAND** | | **R1** | **R2** | **CoMFA** | | **CoMSIA** | | **Docking score** |
| --- | --- | --- | --- | --- | --- | --- | --- | --- | --- |
|  |  |  |  |  | **Pred** | **Res** | **Pred** | **Res** |  |
|  |  |  |  |  |  |  |  |  |  |
|  |  | |  |  | 6.449 |  | 5.629 |  | -9.092 |
|  |  | |  |  | 7.442 |  | 5.629 |  | -9.351 |
|  |  | |  |  | 7.722 |  | 5.629 |  | -7.052 |
|  |  | |  |  | 7.678 |  | 5.629 |  | -9.952 |
|  |  | |  |  | 8.614 |  | 5.629 |  | -9.34 |
|  |  | |  |  | 8.642 |  | 5.629 |  | -7.89 |
|  |  | |  |  | 7.606 |  | 5.629 |  | -9.562 |
|  |  | |  |  | 7.579 |  | 5.629 |  | -9.378 |
|  |  | |  |  | 6.065 |  | 5.629 |  | -8.828 |
|  |  | |  |  | 7.612 |  | 5.634 |  | -9.74 |
|  |  | |  |  | 7.636 |  | 5.629 |  | -9.817 |
|  |  | |  |  | 7.691 |  | 5.629 |  | -8.818 |
|  |  | |  |  | 7.691 |  | 5.629 |  | -9.665 |
|  |  | |  |  | 7.627 |  | 5.630 |  | -8.799 |
|  |  | |  |  | 7.69 |  | 5.629 |  | -9.36 |
|  |  | |  |  | 7.661 |  | 5.630 |  | -9.298 |
|  |  | |  |  | 5.962 |  | 6.154 |  | -8.553 |
|  |  | |  |  | 7.878 |  | 5.630 |  | -8.035 |
|  |  | |  |  | 5.755 |  | 5.626 |  | -8.759 |
|  |  | |  |  | 7.603 |  | 5.629 |  | -9.787 |
|  |  | |  |  | 7.582 |  | 5.629 |  | -6.073 |
|  |  | |  |  | 7.568 |  | 5.629 |  | -8.647 |
|  |  | |  |  | 8.21 |  | 5.629 |  | -10.335 |
|  |  | |  |  | 7.619 |  | 5.629 |  | -9.657 |
|  |  | |  |  | 7.704 |  | 5.629 |  | -8.046 |
|  |  | |  |  | 7.723 |  | 5.629 |  | -9.476 |
|  |  | |  |  | 7.711 |  | 5.629 |  | -9.408 |
|  |  | |  |  | 7.632 |  | 5.629 |  | -9.634 |
|  |  | |  |  | 7.737 |  | 5.629 |  | -9.037 |
|  |  | |  |  | 7.737 |  | 5.629 |  | -9.001 |
|  |  | |  |  | 7.755 |  | 5.629 |  | -9.424 |
|  |  | |  |  | 7.721 |  | 5.629 |  | -9.721 |
|  |  | |  |  | 7.715 |  | 5.629 |  | -9.452 |
|  |  | |  |  | 7.555 |  | 5.629 |  | -12.61 |
|  |  | |  |  | 7.614 |  | 5.629 |  | -9.231 |
|  |  | |  |  | 6.911 |  | 5.629 |  | -8.987 |
|  |  | |  |  | 7.68 |  | 5.629 |  | -12.26 |
|  |  | |  |  | 7.598 |  | 5.629 |  | -6.752 |
|  |  | |  |  | 7.608 |  | 5.625 |  | -9.708 |
|  |  | |  |  | 7.608 |  | 5.629 |  | -9.672 |
|  |  | |  |  | 7.562 |  | 5.629 |  | -9.07 |
|  |  | |  |  | 7.643 |  | 5.629 |  | -9.421 |
|  |  | |  |  | 7.61 |  | 5.629 |  | -9.152 |
|  |  | |  |  | 7.599 |  | 5.629 |  | -8.909 |
|  |  | |  |  | 7.6 |  | 5.629 |  | -8.946 |
|  |  | |  |  | 7.655 |  | 5.622 |  | -9.498 |
|  |  | |  |  | 7.492 |  | 5.629 |  | -6.474 |
|  |  | |  |  | 7.696 |  | 5.629 |  | -9.573 |
|  |  | |  |  | 7.698 |  | 5.629 |  | -9.797 |
|  |  | |  |  | 7.548 |  | 5.629 |  | -8.887 |
|  |  | |  |  | 6.388 |  | 5.629 |  | -6.32 |
|  |  | |  |  | 5.531 |  | 5.630 |  | -6.034 |
|  |  | |  |  | 8.436 |  | 5.636 |  | -6.899 |
|  |  | |  |  | 8.078 |  | 5.629 |  | -8.431 |
|  |  | |  |  | 7.35 |  | 5.629 |  | -8.381 |
|  |  | |  |  | 8.158 |  |  |  | -7.961 |
|  |  | |  |  | 8.323 |  | 5.629 |  | -8.895 |
|  |  | |  |  | 8.245 |  | 5.629 |  | -8.41 |
|  |  | |  |  | 7.72 |  | 5.629 |  | -8.895 |
|  |  | |  |  | 7.479 |  | 5.629 |  | -8.345 |
|  |  | |  |  | 7.985 |  | 5.629 |  | -9.387 |
|  |  | |  |  | 7.825 |  | 5.629 |  | -8.329 |
|  |  | |  |  | 7.219 |  | 5.630 |  | -8.673 |
|  |  | |  |  | 7.828 |  | 5.629 |  | -8.312 |
|  |  | |  |  | 8.16 |  | 5.629 |  | -9.074 |
|  |  | |  |  | 7.873 |  | 5.629 |  | -7.897 |
|  |  | |  |  | 7.626 |  | 5.629 |  | -9.326 |
|  |  | |  |  | 8.009 |  | 5.629 |  | -8.989 |
|  |  | |  |  | 8.305 |  | 5.629 |  | -8.68 |
|  |  | |  |  | 7.766 |  | 5.629 |  | -9.125 |
|  |  | |  |  | 8.091 |  | 5.629 |  | -8.74 |
|  |  | |  |  | 8.42 |  | 5.629 |  | -6.262 |
|  |  | |  |  | 8.391 |  | 5.629 |  | -6.028 |
|  |  | |  |  | 7.857 |  | 5.629 |  | -8.299 |
|  |  | |  |  | 6.055 |  | 5.629 |  | -8.643 |
|  |  | |  |  | 6.433 |  | 5.629 |  | -7.782 |
|  |  | |  |  | 6.067 |  | 5.629 |  | -8.472 |
|  |  | |  |  | 6.064 |  | 5.629 |  | -8.141 |
|  |  | |  |  | 6.303 |  | 5.629 |  | -8.692 |
|  |  | |  |  | 6.571 |  | 5.629 |  | -8.897 |
|  |  | |  |  | 6.34 |  | 5.630 |  | -6.749 |
|  |  | |  |  | 6.341 |  | 5.633 |  | -9.049 |
|  |  | |  |  | 6.344 |  | 5.630 |  | -7.155 |
|  |  | |  |  | 6.611 |  | 5.632 |  | -7.49 |
|  |  | |  |  | 6.502 |  | 5.631 |  | -8.218 |
|  |  | |  |  | 6.342 |  | 5.635 |  | -7.355 |
|  |  | |  |  | 8.614 |  | 5.634 |  | -6.906 |
|  |  | |  |  | 8.368 |  | 5.647 |  | -8.553 |
|  |  | |  |  | 8.477 |  | 5.699 |  | -8.318 |
|  |  | |  |  | 8.482 |  | 5.664 |  | -8.076 |
|  |  | |  |  | 8.523 |  | 5.630 |  | -8.431 |
|  |  | |  |  | 7.875 |  | 5.636 |  | -8.887 |
|  |  | |  |  | 7.247 |  | 5.634 |  | -7.949 |
|  |  | |  |  | 7.951 |  | 5.659 |  | -9.316 |
|  |  | |  |  | 7.751 |  | 5.629 |  | -7.9 |
|  |  | |  |  | 7.857 |  | 5.629 |  | -7.895 |
|  |  | |  |  | 7.92 |  | 5.629 |  | -7.758 |
|  |  | |  |  | 7.573 |  | 5.630 |  | -7.861 |
|  |  | |  |  | 8.413 |  | 5.699 |  | -8.653 |
|  |  | |  |  | 6.488 |  |  |  | -8.801 |

**Table S3:** **Pharmacokinetic properties of 3D-QSAR and in-house library compounds**

| **Compounds** | **Absorption** | **Distribution** | **Metabolism** | | | | | | | **Excretion** | **Toxicity** | **Synthetic**  **accessibility** |
| --- | --- | --- | --- | --- | --- | --- | --- | --- | --- | --- | --- | --- |
|  | **Intestinal absorption**  **(Human)** | **VDss**  **(Human)** | **CYP** | | | | | | | **Total Clearance** | **AMES**  **toxicity** |  |
|  | **Numeric (% absorbed)** | **Numeric (log L/Kg)** | **2D6 substrate** | **3A4 substrate** | **1A2** | **2C19** | **2C9** | **2D6 inhibitor** | **3A4 inhibitor** | **Numeric (log ml/min/kg)** | **Categorical**  **(yes/no)** | **Numeric** |

|  | 63.399 | -0.353 | No | No | No | No | No | No | No | -0.007 | No | 3.45 |
| --- | --- | --- | --- | --- | --- | --- | --- | --- | --- | --- | --- | --- |
|  | 81.885 | -0.308 | No | Yes | Yes | Yes | No | No | Yes | 0.137 | No | 3.55 |
|  | 81.611 | 0.022 | No | Yes | Yes | No | Yes | No | Yes | -0.093 | No | 3.5 |
|  | 74.75 | -0.259 | No | No | Yes | No | No | No | Yes | -0.005 | No | 3.3 |
|  | 76.284 | -0.386 | No | Yes | Yes | No | No | No | Yes | 0.136 | No | 3.56 |
|  | 85.217 | 0.016 | No | Yes | Yes | Yes | No | No | Yes | 0.193 | No | 3.52 |
|  | 86.246 | 0.17 | No | No | Yes | Yes | No | No | Yes | 0.032 | No | 3.37 |
|  | 89.645 | 0.025 | No | Yes | Yes | Yes | Yes | No | Yes | 0.106 | No | 3.3 |
|  | 84.286 | 0.137 | No | Yes | Yes | Yes | Yes | No | Yes | 0.22 | Yes | 3.37 |
|  | 83.217 | 0.026 | No | Yes | Yes | No | No | No | Yes | 0.132 | No | 3.42 |
|  | 84.495 | 0.117 | No | Yes | Yes | Yes | Yes | No | Yes | 0.106 | No | 3.94 |
|  | 84.495 | 0.127 | No | Yes | Yes | Yes | Yes | No | Yes | 0.203 | No | 3.43 |
|  | 88.03 | 0.089 | No | Yes | Yes | Yes | No | No | Yes | 0.231 | No | 3.43 |
|  | 92.89 | -0.062 | No | Yes | Yes | Yes | Yes | No | Yes | 0.103 | No | 3.82 |
|  | 67.29 | -0.387 | No | No | No | No | No | No | No | -0.15 | No | 3.43 |
|  | 87.589 | 0.138 | No | Yes | Yes | Yes | No | No | Yes | 0.177 | No | 3.53 |
|  | 73.043 | -0.216 | No | No | Yes | No | Yes | No | Yes | -0.061 | No | 4.18 |
|  | 86.443 | 0.347 | No | No | No | No | Yes | No | Yes | 0.243 | No | 3.36 |
|  | 86.92 | 0.33 | No | No | Yes | Yes | Yes | No | Yes | 0.207 | No | 3.61 |
|  | 77.43 | 0.158 | No | Yes | Yes | Yes | Yes | No | Yes | 0.106 | No | 3.97 |
|  | 74.992 | 0.944 | No | Yes | Yes | No | No | No | Yes | 0.585 | Yes | 3.67 |
|  | 86.361 | 0.106 | No | No | Yes | Yes | Yes | No | Yes | -0.108 | No | 3.95 |
|  | 89.215 | -0.125 | No | No | Yes | Yes | Yes | No | Yes | 0.007 | No | 3.41 |
|  | 84.221 | 0.094 | No | Yes | Yes | Yes | Yes | No | Yes | 0.186 | No | 3.32 |
|  | 84.392 | 0.087 | No | Yes | Yes | Yes | Yes | No | Yes | 0.217 | No | 4.01 |
|  | 85.226 | 0.073 | No | Yes | Yes | Yes | Yes | No | Yes | 0.117 | No | 3.49 |
|  | 77.121 | -0.059 | No | Yes | Yes | No | No | No | Yes | 0.091 | No | 3.91 |
|  | 88.909 | 0.085 | No | Yes | Yes | Yes | Yes | No | Yes | 0.072 | No | 3.37 |
|  | 78.369 | -0.102 | No | Yes | No | No | Yes | No | Yes | 0.525 | No | 3.44 |
|  | 89.492 | 0.944 | No | Yes | Yes | No | No | No | Yes | 0.585 | Yes | 3.99 |
|  | 87.276 | 0.374 | No | Yes | Yes | Yes | Yes | No | Yes | 0.022 | No | 3.56 |
|  | 88.806 | 0.19 | No | No | Yes | Yes | Yes | No | Yes | 0.163 | No | 3.46 |
|  | 86.567 | 0.006 | No | Yes | Yes | Yes | Yes | No | Yes | 0.143 | No | 3.39 |
|  | **88.633** | **0.373** | **No** | **Yes** | **Yes** | **Yes** | **Yes** | **No** | **Yes** | **-0.059** | **No** | **3.93** |
|  | 72.673 | 0.687 | No | Yes | Yes | No | No | No | Yes | 0.645 | Yes | 3.55 |
|  | 66.886 | -0.306 | No | No | No | No | No | No | Yes | 0.339 | No | 3.97 |
|  | **70.091** | **-0.13** | **No** | **No** | **Yes** | **No** | **No** | **No** | **Yes** | **-0.024** | **No** | **3.66** |
|  | 70.091 | -0.13 | No | No | Yes | No | No | No | Yes | -0.024 | No | 3.42 |
|  | 76.555 | 0.274 | No | No | Yes | Yes | Yes | No | Yes | 0.105 | No | 3.42 |
|  | 83.246 | 0.774 | No | Yes | Yes | Yes | Yes | No | Yes | 0.022 | No | 3.47 |
|  | 87.276 | 0.374 | No | Yes | Yes | Yes | Yes | No | Yes | 0.022 | No | 3.63 |
|  | 88.806 | 0.19 | No | No | Yes | Yes | Yes | No | Yes | 0.163 | No | 3.4 |
|  | 86.567 | 0.006 | No | Yes | Yes | Yes | Yes | No | Yes | 0.143 | No | 3.41 |
|  | 88.633 | 0.373 | No | Yes | Yes | Yes | Yes | No | Yes | -0.059 | No | 3.94 |
|  | 72.673 | 0.687 | No | Yes | Yes | No | No | No | Yes | 0.645 | Yes | 3.58 |
|  | 66.886 | -0.306 | No | No | No | No | No | No | Yes | 0.339 | No | 3.37 |
|  | 70.091 | -0.13 | No | No | Yes | No | No | No | Yes | -0.024 | No | 3.52 |
|  | 70.091 | -0.13 | No | No | Yes | No | No | No | Yes | -0.024 | No | 4.05 |
|  | 76.555 | 0.274 | No | No | Yes | Yes | Yes | No | Yes | 0.105 | No | 3.45 |
|  | 82.276 | 0.454 | No | Yes | Yes | Yes | Yes | No | Yes | 0.022 | No | 3.45 |
|  | 83.931 | 0.222 | No | Yes | Yes | Yes | Yes | No | Yes | 0.102 | No | 3.37 |
|  | 86.468 | 0.256 | No | No | Yes | Yes | Yes | No | Yes | 0.054 | No | 3.02 |
|  | 82.285 | 0.187 | No | Yes | Yes | Yes | Yes | No | Yes | 0.018 | No | 2.91 |
|  | 89.139 | 0.363 | No | Yes | Yes | Yes | Yes | No | Yes | 0.169 | No | 3.54 |
|  | 93.227 | 0.533 | No | No | Yes | No | No | No | Yes | 0.296 | No | 3.47 |
|  | 85.99 | -0.54 | No | No | Yes | Yes | Yes | No | Yes | 0.448 | Yes | 3.1 |
|  | 72.255 | -0.103 | No | Yes | Yes | No | Yes | No | Yes | 0.142 | No | 3.97 |
|  | 89.472 | 0.073 | No | Yes | Yes | Yes | Yes | No | Yes | 0.04 | No | 2.84 |
|  | 74.786 | -0.086 | No | No | Yes | No | Yes | No | Yes | 0.068 | No | 2.95 |
|  | 83.931 | 0.222 | No | Yes | Yes | Yes | Yes | No | Yes | 0.102 | No | 3.01 |
|  | 85.384 | -0.102 | No | No | Yes | No | No | No | Yes | 0.752 | No | 3.4 |
|  | 71.676 | -0.573 | No | Yes | Yes | No | No | No | Yes | -0.189 | No | 3.4 |
|  | 89.673 | -0.102 | No | No | Yes | Yes | No | No | Yes | 0.434 | No | 2.86 |
|  | 90.287 | -0.125 | No | No | Yes | Yes | No | No | Yes | -0.105 | No | 3.05 |
|  | 63.838 | 0.584 | No | Yes | No | No | No | No | No | 0.677 | No | 2.91 |
|  | 83.173 | -0.161 | No | No | Yes | No | No | No | Yes | 0.567 | No | 2.95 |
|  | 86.821 | -0.107 | No | No | Yes | No | No | No | Yes | 0.723 | No | 2.99 |
|  | 68.256 | 0.055 | No | Yes | Yes | No | No | No | No | 0.555 | No | 2.9 |
|  | 80.952 | -0.092 | No | No | Yes | No | No | No | Yes | 0.483 | No | 3.66 |
|  | 85.384 | -0.102 | No | No | Yes | No | No | No | Yes | 0.752 | No | 3.52 |
|  | 83.931 | 0.222 | No | Yes | Yes | Yes | Yes | No | Yes | 0.102 | No | 2.95 |
|  | 86.468 | 0.256 | No | No | Yes | Yes | Yes | No | Yes | 0.054 | No | 2.9 |
|  | 82.285 | 0.187 | No | Yes | Yes | Yes | Yes | No | Yes | 0.018 | No | 2.97 |
|  | 89.139 | 0.363 | No | Yes | Yes | Yes | Yes | No | Yes | 0.169 | No | 3.04 |
|  | 93.227 | 0.533 | No | No | Yes | No | No | No | Yes | 0.296 | No | 3.31 |
|  | 85.99 | -0.54 | No | No | Yes | Yes | Yes | No | Yes | 0.448 | Yes | 3.31 |
|  | 72.255 | -0.103 | No | Yes | Yes | No | Yes | No | Yes | 0.142 | No | 3.27 |
|  | 89.472 | 0.073 | No | Yes | Yes | Yes | Yes | No | Yes | 0.04 | No | 3.35 |
|  | 74.786 | -0.086 | No | No | Yes | No | Yes | No | Yes | 0.068 | No | 2.8 |
|  | 83.931 | 0.222 | No | Yes | Yes | Yes | Yes | No | Yes | 0.102 | No | 3.05 |
|  | 74.09 | -0.17 | No | No | Yes | No | No | No | No | 0.771 | No | 2.82 |
|  | 85.261 | -0.307 | No | No | No | No | No | No | No | 0.336 | No | 3.08 |
|  | 85.261 | -0.307 | No | No | No | No | No | No | No | 0.336 | No | 2.91 |
|  | 79.598 | 0.162 | No | Yes | Yes | Yes | Yes | No | Yes | 0.314 | No | 3.36 |
|  | 87.321 | -0.167 | No | No | Yes | No | No | No | Yes | 0.672 | Yes | 3.36 |
|  | 84.101 | -0.194 | No | No | Yes | No | No | No | Yes | 0.811 | No | 3.54 |
|  | 90.677 | -0.036 | No | No | Yes | Yes | No | No | Yes | 0.713 | Yes | 2.99 |
|  | 90.677 | -0.036 | No | No | Yes | Yes | No | No | Yes | 0.713 | Yes | 2.98 |
|  | 89.133 | -0.176 | No | No | Yes | No | No | No | Yes | 0.403 | No | 2.77 |
|  | 74.09 | -0.17 | No | No | Yes | No | No | No | No | 0.771 | No | 2.77 |
|  | 89.489 | -0.243 | No | Yes | Yes | Yes | Yes | No | Yes | -0.012 | No | 2.87 |
|  | 84.143 | -0.134 | No | No | Yes | No | Yes | No | Yes | 0.534 | No | 3.11 |
|  | 69.77 | -0.1 | No | No | Yes | No | No | No | No | 0.466 | No | 3.03 |
|  | 76.576 | -0.204 | No | Yes | Yes | No | No | No | Yes | 0.373 | No | 2.81 |
|  | 81.738 | -0.093 | No | No | Yes | Yes | Yes | No | Yes | 0.477 | No | 3.16 |
|  | 80.993 | -0.325 | No | No | No | No | No | No | No | -0.235 | No | 3.14 |
|  | 74.979 | -0.124 | No | No | Yes | No | No | No | No | 0.011 | No | 3.28 |
|  | 73.724 | 0.088 | No | No | No | No | No | No | No | 0.66 | No | 3.39 |
|  | 83.363 | -0.331 | No | No | Yes | No | No | No | Yes | -0.185 | No | 3.15 |
|  | 89.489 | -0.243 | No | Yes | Yes | Yes | Yes | No | Yes | -0.012 | No | 2.89 |
